# An image-based protein–ligand binding representation learning framework via multi-level flexible dynamics trajectory pre-training

**DOI:** 10.1093/bioinformatics/btaf535

**Published:** 2025-09-24

**Authors:** Hongxin Xiang, Mingquan Liu, Linlin Hou, Shuting Jin, Jianmin Wang, Jun Xia, Wenjie Du, Sisi Yuan, Xiangzheng Fu, Xinyu Yang, Li Zeng, Lei Xu

**Affiliations:** College of Computer Science and Electronic Engineering, Hunan University, Changsha, Hunan 410082, China; College of Computer Science and Electronic Engineering, Hunan University, Changsha, Hunan 410082, China; College of Computer Science and Electronic Engineering, Hunan University, Changsha, Hunan 410082, China; School of Computer Science & Technology, Wuhan University of Science and Technology, Wuhan, Hubei 430081, China; The Interdisciplinary Graduate Program in Integrative Biotechnology, Yonsei University, Incheon 03722, Korea; School of Engineering, Westlake University, Hangzhou, Zhejiang 310024, China; School of Software Engineering, University of Science and Technology of China, Hefei, Anhui 230026, China; Department of Bioinformatics and Genomics, University of North Carolina at Charlotte, Charlotte, NC 28223, United States; School of Chinese Medicine, Hong Kong Baptist University, Hong Kong SAR 999077, China; College of Computer Science and Electronic Engineering, Hunan University, Changsha, Hunan 410082, China; Department of AIDD, Shanghai Yuyao Biotechnology Co., Shanghai 200000, China; School of Electronic and Communication Engineering, Shenzhen Polytechnic University, Shenzhen 518055, China

## Abstract

**Motivation:**

Accurate prediction of protein–ligand binding (PLB) relationships plays a crucial role in drug discovery, which helps identify drugs that modulate the activity of specific targets. Traditional biological assays for measuring PLB relationships are time consuming and costly. In addition, models for predicting PLB relationships have been developed and widely used in drug discovery tasks. However, learning more accurate PLB representations is essential to meet the stringent standards required for drug discovery.

**Results:**

We propose an image-based PLB representation learning framework, called ImagePLB, which equips ligand representation learner (LRL) and protein representation learner (PRL) to accept 3D multi-view ligand images and protein graphs as input, respectively, and learns rich interaction information between ligand and protein through a binding representation learner (BRL). Considering the scarcity of protein–ligand pairs, we further propose a multi-level next trajectory prediction (MLNTP) task to pre-train ImagePLB on the 4D flexible dynamics trajectory of 16 972 complexes, including ligand level, protein level, and complex level, to learn information related to trajectories. Besides, by introducing trajectory regularization (TR), we effectively alleviate the problem of high (even almost identical) feature similarity caused by adjacent trajectories. Compared with the current state-of-the-art methods, ImagePLB has achieved competitive improvements on PLB-related prediction tasks, including protein–ligand affinity and efficacy prediction tasks. This study opens the door to the image-based PLB learning paradigm.

**Availability and implementation:**

All data and implementation details of code can be obtained from https://github.com/HongxinXiang/ImagePLB.

## 1 Introduction

With the development of deep learning (DL), more and more researchers have begun to turn to DL methods to solve problems in drug discovery, hoping to greatly accelerate the process of drug development (Hou *et al.* 2024, Xiang *et al.* 2024b). Learning effective representations of protein and ligand binding is crucial in various tasks of drug discovery (Zhang *et al.* 2022, Fu *et al.* 2024, Hong *et al.* 2024), such as affinity prediction (Öztürk *et al.* 2018, Rube *et al.* 2022, Mou *et al.* 2023), molecular docking (Zhang *et al.* 2023b, Cao *et al.* 2024), and virtual screening (Tanrikulu and Schneider 2008, Nguyen-Vo *et al.* 2024), which measures the strength of the interaction between a protein and a ligand. We summarize existing related work in Appendix A, available as supplementary data at *Bioinformatics* online. Given that atoms in proteins and ligands can be naturally represented as vertices in a graph, a large number of graph neural networks (GNNs)-based methods (Somnath *et al.* 2021, Wu *et al.* 2022, Wang *et al.* 2023) have recently been proposed for protein–ligand binding (PLB) representation learning and have shown promising performance.

**Figure 1.**
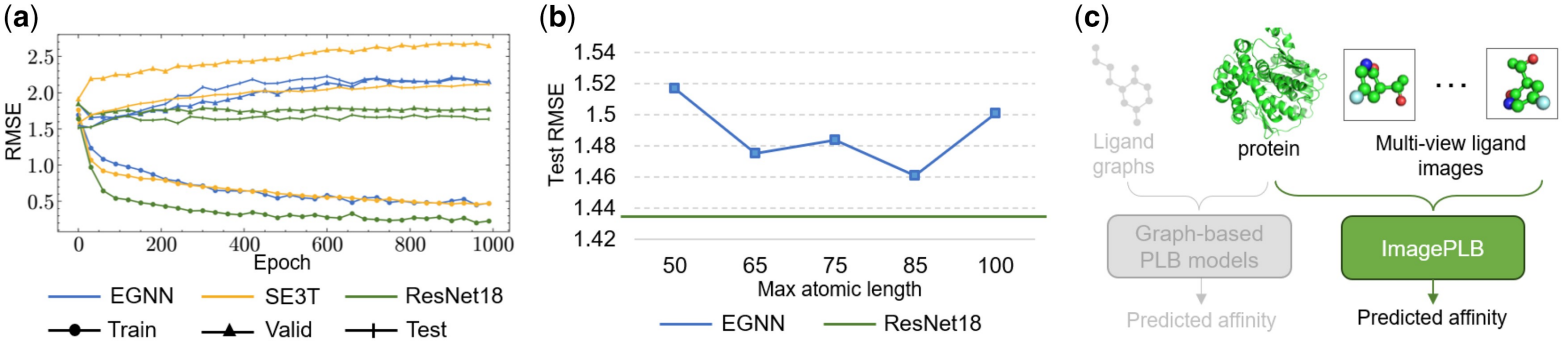
(a) Performance of EGNN, SE(3)-Transformer, and ResNet18 on train, validation, and test sets of PDBbind-30 for 1000 epochs. PDBbind-30 represents splitting dataset to train/valid/test sets with 30% sequence identity. (b) Performance of EGNN and ResNet18 on PDBbind-30 with different maximum atomic length. The test results are picked with the best validation performance. (c) The proposed protein–ligand binding (PLB) representation learning framework (called ImagePLB), which is different from the previous graph-based PLB models and uses images to represent ligands.

However, we find that existing graph-based methods face two inherent challenges: (i) limited in capturing high-quality PLB representations; and (ii) being sensitive to the maximum atomic length. Here, we take PDBbind dataset (Liu *et al.* 2017) with the data splitting of 30% sequence identity (Somnath *et al.* 2021) as an example. Figure 1a shows the performance of EGNN (Satorras *et al.* 2021) and SE3T (SE(3)-Transformer) (Fuchs *et al.* 2020) on train, valid, and test sets for 1000 epochs. We found that as the number of epochs continued to increase, the performance of EGNN and SE3T on the training set improved only slightly, converging to a root mean squared error (RMSE) of around 0.5. More importantly, we found that the performance of EGNN and SE3T on the validation set and test set is obviously inversely proportional to the performance on the training set. In particular, for SE3T, at the 1000th epoch, the performance difference between the training set and the validation set is as high as 5.6 times. As we all know, the performance of a model on the training set reflects its fitting ability (low performance on the training set means that the model has an underfitting problem), while the performance on the validation set and test set indicates its generalization ability to unknown samples. Generally, there are two common model selection strategies: (i) selection based on the best performance on the training set, which ensures that the model has learned enough knowledge from the training set; and (ii) selection based on the best performance on the validation set, which ensures that the model has sufficient generalization ability. If the model is selected based on the best validation performance, the model is underfit on the training set, which is because at the epoch with the best performance on the validation set, the performance of the model on the training set is low. If the model is selected based on the best training performance, the model does not have the ability to generalize, which is because in the epoch with the best performance on the training set, the performance of the model on the validation set and test set is low. This phenomenon indicates that graph-based PLB models are limited in capturing high-quality PLB representation in the affinity prediction task.

Furthermore, ligands are composed of multiple atoms, such as C, N, O, etc., and graph-based PLB needs to treat each atom in the ligand as a node in the graph. Obviously, the memory and computational complexity of graph-based models are related to the number of atoms. We define the maximum number of atoms θ as the upper limit on the number of atoms that can be contained in a molecule. If the number of atoms exceeds θ, the molecule will be truncated (some atoms will be removed), while for molecules with less than θ atoms, they will be filled to meet the maximum number of atoms. Therefore, some methods (Wu *et al.* 2022) maintain stable computational efficiency by specifying the maximum number of atoms in the ligand. However, we found that the graph-based PLB model is sensitive to the maximum atom length θ because adjusting θ will cause the performance of the model to fluctuate. In detail, Fig. 1b shows the performance of EGNN with different maximum atomic length and we can clearly see the change in performance when the atomic length changes with the maximum performance difference reaching 3.8%. We provide a more detailed motivation analysis in Appendix B, available as supplementary data at *Bioinformatics* online.

To overcome the challenges mentioned above, we propose to use multi-view ligand images (Xiang *et al.* 2024a) to learn the PLB representation in Fig. 1c, called ImagePLB. As shown in Fig. 1a, the ImagePLB equipped with ResNet18 (He *et al.* 2016) achieves a lower RMSE performance of about 0.2 on the training data, improving more than two times higher than EGNN and SE3T on performance, and more stable RMSE performance on the validation set (variance of 0.026) and test set (variance of 0.041), improving more than seven times and four times higher than EGNN and SE3T on the validation set and test set. This proves that the proposed ImagePLB has stronger fitting ability and more stable generalization ability. The ImagePLB does not encode the atoms in the ligand, so it has the inherent advantage of being insensitive to the maximum number of atoms.

In addition, labeling the interactions between proteins and ligands is expensive and time consuming, relying on biological wet experiments, which limits the number of PLB pairs (Gorgulla *et al.* 2020, Sun *et al.* 2021). To better help the model learn more abundant interactions between proteins and ligands in limited data, we introduce a transformer-based binding representation learner (Vaswani *et al.* 2017), which learns dual interactions between protein and ligand pairs to enhance binding representation. The interaction between proteins and ligands is a dynamic process and their conformations are constantly adjusted by the influence of each other’s force fields (Karplus and Petsko 1990). Considering the rise of self-supervised learning (Liu *et al.* 2023), we consider the concept of time in the interaction process and introduce 4D flexible dynamics trajectories (Siebenmorgen *et al.* 2024) to pre-train ImagePLB, called ImagePLB-P, with a multi-level next trajectory prediction pre-task, including ligand, protein, and complexlevel, to learn information related to trajectories.

## 2 Materials and methods

### 2.1 Problem definition

Let the ligand–protein complexes and corresponding ground-truth labels on downstream tasks are ${\left\{C_{i}=(L_{i},P_{i})\right\}}_{i=1}^{n}$ and ${\left\{y_{i}\right\}}_{i=n}^{n}\in R^{1}$, respectively, where L and P represent ligands and proteins. The ligand and protein are represented as a 3D image $L_{i}\in R^{V\times H\times W\times 3}$ and a geometric graph $P_{i}\in R^{n_{P}\times 3}$, respectively, where *V*, *H*, *W*, 3 represent the number of views, height, width, channel of the image, and $n_{P},3$ represents the number of amino acids and the corresponding 3D coordinates in the protein. Different from existing methods, we define the PLB problem as a cross-modal matching problem between images and geometric graphs. Therefore, image-based ligand encoder Enc${\;}^{L}$ and graph-based protein encoder Enc${\;}^{P}$ are used to extract ligand feature $F^{L}\in R^{d^{L}}$ and protein feature $F^{P}\in R^{d^{P}}$, respectively. Then, a binding representation learning model Enc${\;}^{F}$ is used to fuse features of the ligand and the protein to obtain $F^{B}=$Enc${\;}^{F}(F^{L},F^{P})$. Finally, a predictor *g* is used to obtain the prediction result $\hat{y}=g(F^{B})$ for the downstream task.

### 2.2 ImagePLB

**Figure 2.**
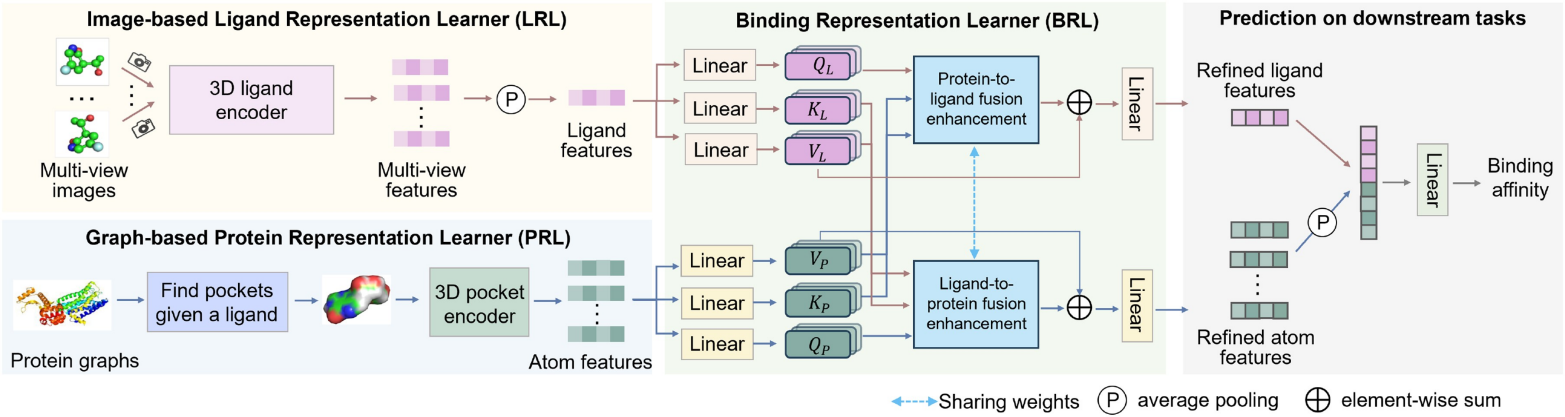
Overview of the proposed ImagePLB. LRL and PRL are used to extract ligand features and α-C atom features of protein, respectively. BRL enhances the interaction between ligand and protein and extracts refined ligand features and refined atom features. Finally, refined ligand features and refined atom features are concatenated together and input into the predictor to predict the results of downstream tasks.

As shown in Fig. 2, the ImagePLB pipeline includes the following steps. First, the image-based ligand representation learner (LRL) and graph-based protein representation learner (PRL) extract features from the ligand image and protein geometry graph, respectively. Then, the binding representation learner (BRL) is used to learn the interaction information between the ligand and the protein. Finally, a predictor is used to evaluate the downstream task.

#### 2.2.1 Ligand representation learner

We generate multi-view 3D images L of the ligand by using PyMol (DeLano *et al.* 2002) with command *bg_color white; set stick_ball, on; set stick_ball_ratio, 3.5; set stick_radius, 0.15; set sphere_scale, 0.2; set valence, 1; set valence_mode, 0; set valence_size, 0.1*. Here, we set the number of views to V=4, which includes the original view, a 180° rotation along the *x*-axis, a 180° rotation along the *y*-axis, and a 180° rotation along the *z*-axis. See Appendix C, available as supplementary data at *Bioinformatics* online, for more details on multi-view images. Then, we forward L to a 3D ligand encoder Enc${\;}^{L}$ to obtain multi-view features $F^{L}\in R^{n\times V\times d^{L}}$. We use ResNet18 as Enc${\;}^{L}$ and its weights are initialized with ImageNet (Deng *et al.* 2009). Finally, we perform average pooling on *V* to extract the ligand feature $F^{L}\in R^{n\times d^{L}}$.

#### 2.2.2 Protein representation learner

For a given complex conformation, we first search for pocket regions on the protein based on the ligand. We select only amino acids within 6 Å of the ligand atoms and use the coordinates of α-C atom to construct geometric graph of the pocket $P\in R^{n\times n_{P}\times 3}$. Since the number of α-C atoms $n_{P}$ in different pockets is different, we set the maximum atomic length $n_{\alpha }$ to obtain $P\in R^{n\times n_{\alpha }\times 3}$, which means that excess atoms will be truncated and insufficient atoms will be supplemented with meaningless characters. We use $m\in R^{n\times n_{\alpha }}$ to mark which pocket an atom in P comes from. Next, we input P and *m* into the 3D pocket encoder to extract atomic-level features $F^{\alpha }\in R^{n\times n_{\alpha }\times d^{P}}$.

#### 2.2.3 Binding representation learner

BRL aims to learn rich interaction information between $F^{L}$ and $F^{\alpha }$. We first linearly map $F^{L}$ to a feature ${\hat{F}}^{L}\in R^{n\times d^{P}}$ with a same dimension as $F^{\alpha }$. For convenience, we concatenate the atoms of all pockets in $F^{\alpha }$ to obtain ${\hat{F}}^{\alpha }\in R^{n_{a}\times d^{P}}$ and $\hat{m}\in R^{n_{a}}$, where $n_{a}$ represents the number of atoms in all *n* pockets and we can find the pocket to which the atom belongs according to $\hat{m}$. Then, we define six linear layers $f_{L,Q}$, $f_{L,K}$, $f_{L,V}$, $f_{P,Q}$, $f_{P,K}$, $f_{P,V}$ with input dimension of $d^{P}$ and output dimension of $d^{o}$ to extract features $Q_{L}=f_{L,Q}({\hat{F}}^{L})$, $K_{L}=f_{L,K}({\hat{F}}^{L})$, $V_{L}=f_{L,V}({\hat{F}}^{L})$, $Q_{P}=f_{P,Q}({\hat{F}}^{\alpha })$, $K_{P}=f_{P,K}({\hat{F}}^{\alpha })$, $V_{P}=f_{P,V}({\hat{F}}^{\alpha })$, respectively, where $Q_{L},K_{L},V_{L}\in R^{n\times d_{o}}$ and $Q_{P},K_{P},V_{P}\in R^{n_{a}\times d_{o}}$. These features are further represented in the form of $n_{h}$ heads: ${\hat{Q}}_{L},{\hat{K}}_{L},{\hat{V}}_{L}\in R^{n_{h}\times n\times {\hat{d}}_{o}}$ and ${\hat{Q}}_{P},{\hat{K}}_{P},{\hat{V}}_{P}\in R^{n_{h}\times n_{a}\times {\hat{d}}_{o}}$, where $d_{o}=n_{h}\times {\hat{d}}_{o}$.

#### 2.2.4 Dual fusion enhancement

Given the representations ${\hat{Q}}_{L},{\hat{K}}_{L},{\hat{V}}_{L}$ from the ligand image and the representations ${\hat{Q}}_{P},{\hat{K}}_{P},{\hat{V}}_{P}$ from the protein graph and the corresponding mask matrix $\hat{m}$, we use Protein-to-Ligand Fusion Enhancement and Ligand-to-Protein Fusion Enhancement to enhance each other’s cross-modal interaction information. We take protein-to-ligand fusion enhancement as an example to illustrate this process in detail. First, we use ${\hat{Q}}_{L}$ and ${\hat{K}}_{P}$ to compute multi-head attention ϕ, which is formalized as:


(1) $$\phi =\frac{{\hat{Q}}_{L}⊗{\hat{K}}_{P}\cdot m}{\sqrt[{2}]{{\hat{d}}_{o}}}\in R^{n_{h}\times n\times n_{a}}$$

where ⊗ and · denote matrix multiplication and Hadamard product, respectively. To make the softmax function ignore zero values in the attention, we set the zero values in ϕ to a very small number (−9e10) and obtain the probability value $\hat{\phi }=\mathrm{Softmax}(\phi )$. Therefore, we can fuse the protein context to the features of the ligand as follows:


(2) $$H={\hat{V}}_{L}+\hat{\phi }⊗{\hat{V}}_{P}\in R^{n_{h}\times n\times {\hat{d}}_{o}}$$

We resize the dimension of H to $n\times d_{o}$ and pass it through a fully connected layer to obtain the refined ligand features $H^{L}\in R^{n\times d_{o}}$. Similarly, we used ligand-to-protein fusion enhancement to obtain refined atom features $H^{P}\in R^{n_{a}\times d_{o}}$.

Finally, we perform average pooling on the atoms of $H^{P}$ to obtain refined pocket features ${\hat{H}}^{P}\in R^{n\times d_{o}}$ and concatenate it with $H^{L}$ and input it into a predictor to obtain the predicted logits $\hat{y}$.

### 2.3 Multi-level next trajectory prediction

Previous pre-training methods focused on understanding the static conformations of ligands and proteins (Zhou *et al.* 2023, Gao *et al.* 2023). However, as we all know, the binding of ligands and proteins is a dynamic process and the conformational changes of ligands and proteins during the binding process can promote the model to learn rich binding information (Lecina *et al.* 2017). Here, we propose a multi-level next trajectory prediction (MLNTP) task to self-supervised pre-train the proposed ImagePLB. We use 10 nanoseconds (ns) of complex dynamics trajectory and discard the first 2 ns of complex trajectory (the first 2 ns are discarded as equilibration phase (Siebenmorgen *et al.* 2024)). We acquire 100 video frames from the remaining 8 ns. Each frame is equally spaced (interval is 0.08 ns).

**Figure 3.**
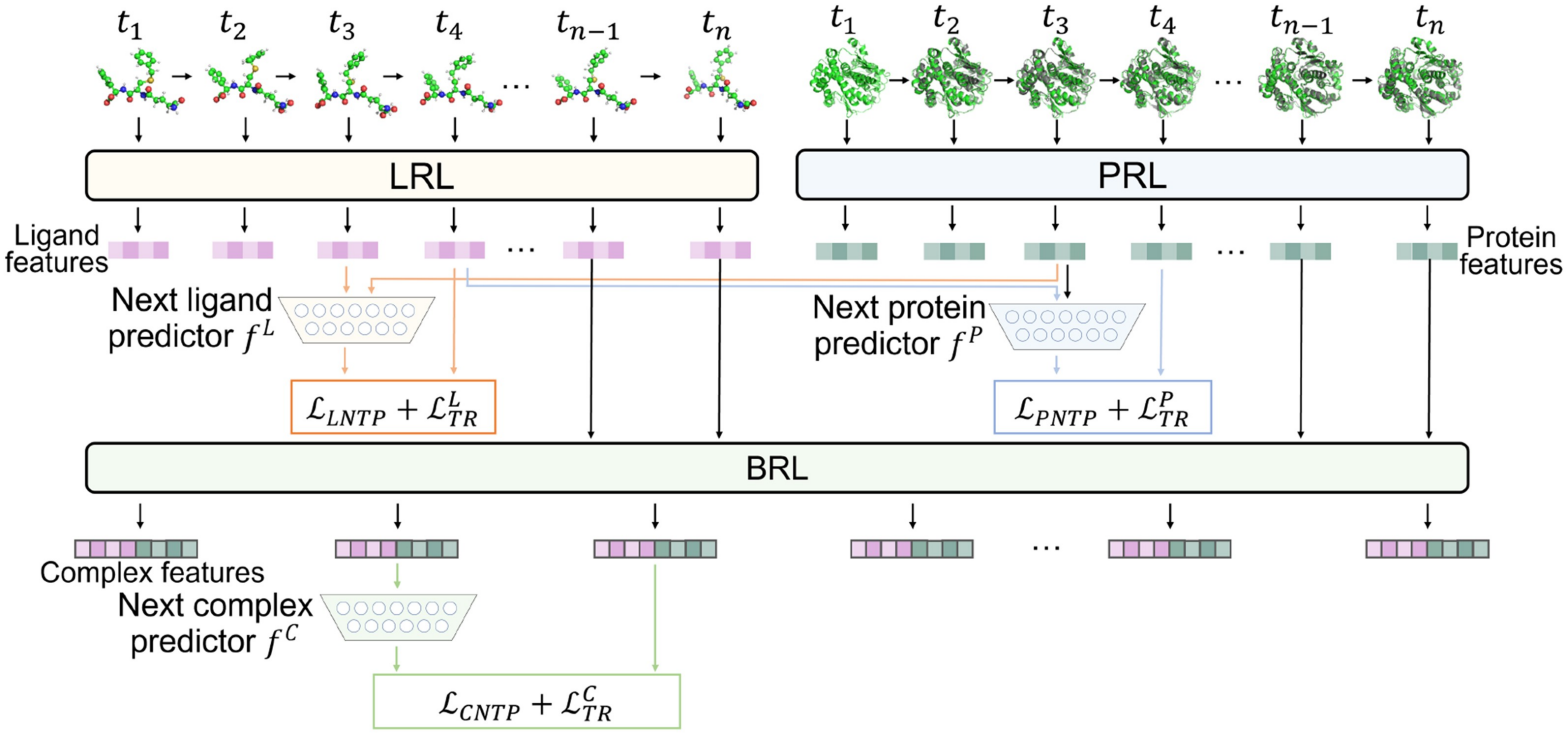
Overview of the proposed multi-level next trajectory prediction (MLNTP). We predict the trajectory at $t_{n}$ from three levels (ligand, protein, and complex) based on the trajectory at $t_{n-1}$. In protein, in order to better show the differences between adjacent frames, we use black areas to show the protein in the previous frame.

As show in Fig. 3, the main idea of MLNTP is to predict the conformations of the ligand, protein, and complex at time t+1 given the binding conformation of the *i*th ligand–protein complex $C_{i,t}=\{L_{i,t},P_{i,t}\}$ at time *t*. We first extract ligand features $\{F_{i,t}^{L},F_{i,t+1}^{L}\}\in R^{d^{L}}$, pocket features $\{F_{i,t}^{P},F_{i,t+1}^{P}\}\in R^{d^{P}}$ (which is obtained by performing average pooling on the atoms of $F^{\alpha }$), refined ligand features $\{H_{i,t}^{L}H_{i,t+1}^{L}\}\in R^{d^{o}}$ and refined pocket features $\{{\hat{H}}_{i,t}^{P},{\hat{H}}_{i,t+1}^{P}\}\in R^{d^{o}}$ from $C_{i,t}$ and $C_{i,t+1}$. Next, we obtain the feature of the complex as follows:


(3) $$F_{i,t}^{C}=H_{i,t}^{L}+{\hat{H}}_{i,t}^{P};\;F_{i,t+1}^{C}=H_{i,t+1}^{L}+{\hat{H}}_{i,t+1}^{P}$$

Subsequently, we define three conditional predictors $f^{L}$, $f^{P}$, $f^{C}$ with the same structure to learn the dynamic trajectory information at the molecule level, protein level, and complex level, respectively. The conditional predictor can be formalized as f(x,condition)=network([x,linear(condition)]), which represents predicting the next frame trajectory of *x* under the *condition*. Therefore, the prediction result of the next frame trajectory can be obtained as follows:


(4) $$\begin{array}{c} F_{i,t+1}^{L,*}=f^{L}(F_{i,t}^{L},F_{i,t}^{P});\;F_{i,t+1}^{P,*}=f^{P}(F_{i,t}^{P},F_{i,t}^{L});\\ F_{i,t+1}^{C,*}=f^{C}(F_{i,t}^{C}) \end{array}$$

Here, we use L1 loss to align the predicted trajectory with the true trajectory. The loss function can be formalized as:


(5) $$L_{\mathrm{LNTP}}=L1(F_{i,t+1}^{L,*},F_{i,t+1}^{L})$$

(6) $$L_{\mathrm{PNTP}}=L1(F_{i,t+1}^{P,*},F_{i,t+1}^{P})$$

(7) $$L_{\mathrm{CMTP}}=L1(F_{i,t+1}^{C,*},F_{i,t+1}^{C})$$

### 2.4 Trajectory regularization

We find that simply introducing the trajectory prediction task easily leads to the model collapse problem, which is caused by the fact that adjacent trajectories are too similar (or even identical). Therefore, we introduce trajectory regularization to ensure the differences between adjacent trajectories, which can be formalized as:


(8) $$\begin{array}{c} L_{TR}^{L}=\text{exp}(|F_{i,t}^{L},F_{i,t+1}^{L}|);\;L_{TR}^{P}=\text{exp}(|F_{i,t}^{P},F_{i,t+1}^{P}|);\\ L_{TR}^{C}=\text{exp}(|F_{i,t}^{C},F_{i,t+1}^{C}|) \end{array}$$

where |·| represents calculating the MSE distance. Therefore, the final pre-training loss is as follows:


(9) $$\begin{array}{c} L_{\mathrm{Pretrain}}=\lambda_{L}L_{\mathrm{LNTP}}+(1-\lambda_{L})L_{TR}^{L}\\ +\lambda_{P}L_{\mathrm{PNTP}}+(1-\lambda_{P})L_{TR}^{P}\\ +\lambda_{C}L_{\mathrm{CMTP}}+(1-\lambda_{C})L_{TR}^{C} \end{array}$$

where $\lambda_{*}=\frac{L_{*\mathrm{NTP}}}{L_{*\mathrm{NTP}}+L_{TR}^{*}}$ and * represents {L,P,C}. The purpose of $\lambda_{*}$ is to weigh the similarities and differences between adjacent trajectories.

## 3 Results

### 3.1 ImagePLB framework

As shown in Fig. 2, our research introduces an image-based PLB representation learning framework, called ImagePLB. ImagePLB first uses two learners, image-based LRL and graph-based PRL, to learn the information of ligands and proteins, respectively. In LRL, ligand conformations are rendered into multi-view images and 3D ligand encoder is used to obtain ligand features. In PRL, protein conformations are converted into pockets and 3D pocket encoder is used to obtain pocket features. Subsequently, ImagePLB uses a BRL to enhance the interaction information between ligands and pockets. Finally, we concatenate them and input them into a predictor to predict the results of downstream tasks.

Next, we propose a pre-training strategy, called multi-level next trajectory pre-training (MLNTP), to train ImagePLB. We call the pre-trained ImagePLB ImagePLB-P. As shown in Fig. 3, we input the flexible dynamics trajectory into ImagePLB and predict the conformational change at time t+1 given the conformation of the ligand, protein, or complex at time *t*. See the Section 2 for more details.

### 3.2 Experimental settings

#### 3.2.1 Datasets

We use MISATO (Siebenmorgen *et al.* 2024) as a pre-training dataset for ImagePLB, which combines quantum mechanical properties of small molecules and associated molecular dynamics simulations (accumulating over 170 μs) of ∼20 000 experimental protein–ligand complexes with extensive validation of experimental data. To verify the effectiveness of ImagePLB, we use four benchmarks (Townshend *et al.* 2021) commonly used in protein–ligand interaction representation learning, namely PDBbind-30, PDBbind-60, PDBbind-scaffold (Liu *et al.* 2015) from PDBbind v2019 (Liu *et al.* 2017) and LEP (Ligand Efficacy Prediction) (Friesner *et al.* 2004). The task of PDBbind is to predict p*K* = −log(*K*), where *K* is the binding affinity in molar units, given a complex of a protein and a ligand. We used 30% and 60% sequence identity thresholds and scaffold to restrict homologous ligands or proteins and divided the complexes into training, validation, and test sets. Especially, in PDBbind-30 and PDBbind-60, the proteins in the training/validation/test sets do not overlap and this setting evaluates the generalization ability of the model on proteins. In PDBbind-scaffold, the training/validation/test sets are split according to the scaffold of the ligand, which means that the ligands in different sets do not overlap. We provide the details of PDBbind and the corresponding splits in Appendix D, available as supplementary data at *Bioinformatics* online. The task of LEP is to predict whether a molecule binding to a structure will become an activator of protein function, which is a binary classification task.

#### 3.2.2 Evaluation protocol

Following Wu *et al.* (2022), on the PDBbind datasets, we use RMSE, Pearson correlation coefficient, and Spearman correlation coefficient as evaluation metrics. On the LEP dataset, we use accuracy (ACC), area under the receiver operating characteristic curve (AUROC), and area under the precision–recall curve (AUPRC) as evaluation metrics. We use the validation set to select the results for the test set and report the mean and variance of all experimental results using three different random seeds ranging from 0 to 2.

#### 3.2.3 Baselines

We selected a large number of the latest baselines for comparative experiments. For non-pretrained models, we selected four different types of methods, including *sequence-based methods* such as DeepDTA (Öztürk *et al.* 2018), SSA (Bepler and Berger 2019), TAPE (Rao *et al.* 2019), ProtTrans (Ahmed *et al.* 2020), and SSM-DTA (Pei *et al.* 2023), *surface-based methods* such as MaSIF (Gainza *et al.* 2020) and HoloProt (Somnath *et al.* 2021), *voxel-based method* such as 3DCNN (Townshend *et al.* 2021), and *graph-based methods* such as IEConv, ProtMD (Wu *et al.* 2022), and LEFTNet (Du *et al.* 2023). For the pre-training methods, we selected graph-based methods, including ProtMD-P (Wu *et al.* 2022), Uni-Mol (Zhou *et al.* 2023), GeoSSL-DDM (Liu *et al.* 2023), and DrugCLIP (Gao *et al.* 2023), as our comparison methods. See Appendix E, available as supplementary data at *Bioinformatics* online, for details of baselines.

#### 3.2.4 Implementation details

We use ResNet18 and EGNN as 3D ligand encoder and 3D pocket encoder to extract 512-dimensional multi-view features and 100-dimensional α-C atom features, respectively. In EGNN, we set the maximum atom length of α-C to 100, which means that when the number of atoms in a pocket is greater than 100, the extra atoms will be truncated. In the benchmark datasets, the maximum number of atoms in a pocket does not exceed 100 (see Appendix F, available as supplementary data at *Bioinformatics* online, for detailed distribution of datasets). We pre-train ImagePLB on the MISATO dataset for 30 epochs with batch size 128 and learning rate 0.001 to obtain ImagePLB-P. We discuss the computational cost of this pre-training in detail in Appendix G, available as supplementary data at *Bioinformatics* online. On the downstream tasks (PDBbind and LEP), we conducted grid search with different learning rates, dropout rates, batch sizes, and epochs. See Appendix H, available as supplementary data at *Bioinformatics* online, for detailed hyperparameter search ranges. Experiments are performed on an Ubuntu 18.04 server with an AMD EPYC 7542 32-Core Processor, eight NVIDIA GeForce RTX 4090, and 512 GB of RAM.

### 3.3 Main results

We first evaluate ImagePLB and ImagePLB-P on PDBbind-30, PDBbind-60, and PDBbind-Scaffold against nine non-pretrained baselines and four pretrained baselines. As shown in Table 1, we find that ImagePLB outperforms all non-pretrained models on RMSE, Pearson, and Spearman metrics, which demonstrates the effectiveness of the ImagePLB framework. After pre-training, ImagePLB-P further improves the performance of ImagePLB with an average improvement of 2.2%, especially on PDBbind-30 and PDBbind-60, outperforming four existing pre-training methods. Next, we turn to evaluating ImagePLB and ImagePLB-P on the LEP task. As shown in Table 2, ImagePLB outperforms existing non-pretrained models with 15.8% improvement on AUROC and 30.6% improvement on AUPR and ImagePLB-P outperforms existing pretrained methods with 5.5% improvement on AUROC and 13.8% improvement on AUPR. Overall, ImagePLB is a very competitive model and changes the learning paradigm of PLB representation learning.

**Table 1.** The RMSE, Pearson, Spearman of different methods on PDBbind datasets with 30%, 60%, and scaffold splittings.^a^

Model	Sequence identity (30 %)			Sequence identity (60 %)			Scaffold		
	RMSE ↓	Pearson ↑	Spearman ↑	RMSE ↓	Pearson ↑	Spearman ↑	RMSE ↓	Pearson ↑	Spearman ↑
DeepDTA	1.866 ± 0.080	0.472 ± 0.022	0.471 ± 0.024	1.762 ± 0.261	0.666 ± 0.012	0.663 ± 0.015	1.908 ± 0.145	0.384 ± 0.014	0.387 ± 0.016
SSA	1.985 ± 0.006	0.165 ± 0.006	0.152 ± 0.024	1.891 ± 0.004	0.249 ± 0.006	0.275 ± 0.008	1.864 ± 0.009	0.269 ± 0.002	0.285 ± 0.019
TAPE	1.890 ± 0.035	0.338 ± 0.044	0.286 ± 0.124	1.633 ± 0.016	0.568 ± 0.033	0.571 ± 0.021	1.680 ± 0.055	0.487 ± 0.029	0.462 ± 0.051
ProtTrans	1.544 ± 0.015	0.438 ± 0.053	0.434 ± 0.058	1.641 ± 0.016	0.595 ± 0.014	0.588 ± 0.009	1.592 ± 0.009	0.398 ± 0.027	0.409 ± 0.029
MaSIF	1.484 ± 0.018	0.467 ± 0.020	0.455 ± 0.014	1.426 ± 0.017	0.709 ± 0.008	0.701 ± 0.011	1.583 ± 0.132	0.416 ± 0.111	0.412 ± 0.126
3DCNN	1.429 ± 0.042	0.541 ± 0.029	0.532 ± 0.033	1.450 ± 0.024	0.716 ± 0.008	0.714 ± 0.009	–	–	–
IEConv	1.554 ± 0.016	0.414 ± 0.053	0.428 ± 0.032	1.473 ± 0.024	0.667 ± 0.011	0.675 ± 0.019	1.592 ± 0.012	0.365 ± 0.024	0.373 ± 0.019
HoloProt	1.464 ± 0.006	0.509 ± 0.002	0.500 ± 0.005	–	–	–	1.516 ± 0.014	0.491 ± 0.016	0.493 ± 0.014
ProtMD	1.541 ± 0.030	0.542 ± 0.048	0.527 ± 0.041	1.572 ± 0.013	0.654 ± 0.004	0.647 ± 0.009	1.530 ± 0.002	0.493 ± 0.012	0.477 ± 0.008
LEFTNet	**1.398 ± 0.008**	**0.575 ± 0.007**	**0.566 ± 0.003**	1.591 ± 0.007	0.624 ± 0.003	0.633 ± 0.003	1.571 ± 0.010	0.441 ± 0.010	0.435 ± 0.007
ImagePLB	1.413 ± 0.008	0.562 ± 0.015	0.551 ± 0.012	**1.396 ± 0.041**	**0.730 ± 0.018**	**0.733 ± 0.021**	**1.494 ± 0.015**	**0.512 ± 0.019**	**0.511 ± 0.028**
SSM-DTA	1.543 ± 0.015	0.461 ± 0.008	0.471 ± 0.010	–	–	–	–	–	
GeoSSL-DDM	1.451 ± 0.030	0.577 ± 0.020	0.572 ± 0.010	–	–	–	–	–	–
ProtMD-P	1.389 ± 0.020	0.587 ± 0.004	0.586 ± 0.006	1.498 ± 0.035	0.679 ± 0.014	0.670 ± 0.020	**1.506 ± 0.014**	0.475 ± 0.024	0.464 ± 0.020
Uni-Mol	1.562 ± 0.018	0.443 ± 0.004	0.444 ± 0.007	1.506 ± 0.019	0.678 ± 0.013	0.689 ± 0.010	1.540 ± 0.008	0.479 ± 0.005	0.490 ± 0.006
DrugCLIP	1.554 ± 0.013	0.444 ± 0.017	0.447 ± 0.017	1.531 ± 0.063	0.671 ± 0.020	0.666 ± 0.041	1.547 ± 0.006	0.479 ± 0.012	0.484 ± 0.010
ImagePLB-P	**1.352 ± 0.021**	**0.606 ± 0.012**	**0.601 ± 0.016**	**1.379 ± 0.008**	**0.737 ± 0.004**	**0.735 ± 0.005**	1.513 ± 0.032	**0.499 ± 0.031**	**0.511 ± 0.027**

a The bold values indicate the best results, and values with underlines indicate the second-best results. Before ImagePLB, there is a comparison of methods without pre-training on molecules and proteins, and after ImagePLB, there is a comparison of pre-training methods. These results are reported for three experimental runs with three random seeds.

**Table 2.** The ACC, AUROC, and AUPRC of different methods on the ligand efficacy prediction task.^a^

	ACC ↑	AUROC ↑	AUPRC ↑
3DCNN	–	0.589 ± 0.020	0.483 ± 0.037
3DGCN	–	0.681 ± 0.062	0.598 ± 0.135
Cormorant	–	0.663 ± 0.100	0.551 ± 0.121
DeepDTA	–	0.696 ± 0.021	0.550 ± 0.024
SchNet	0.615 ± 0.031	0.722 ± 0.017	0.703 ± 0.011
PaiNN	0.628 ± 0.025	0.711 ± 0.020	0.732 ± 0.007
Equiformer	0.654 ± 0.028	0.735 ± 0.028	0.726 ± 0.032
LEFTNet	0.704 ± 0.043	0.766 ± 0.019	0.730 ± 0.032
ImagePLB	**0.758 ± 0.005**	**0.806 ± 0.043**	**0.781 ± 0.026**
ProtMD-P	–	0.742 ± 0.039	0.724 ± 0.041
GeoSSL-DDM	–	0.776 ± 0.030	0.694 ± 0.060
ImagePLB-P	**0.755 ± 0.058**	**0.819 ± 0.055**	**0.790 ± 0.026**

a The bold values indicate the best results. The results are reported for three experimental runs with three random seeds.

To further evaluate the generalization ability of the model on protein and ligand pairs, we further increase the restriction of ligand nonoverlap based on PDBbind-30 and obtain a new dataset PDBbind-30-nonoverlap. Specifically, we remove complexes with overlapping proteins or ligands in the training, validation, and test sets. In the PDBbind-30-nonoverlap dataset, there is no overlap in the proteins or ligands in the training set (3313 complexes), validation set (461 complexes), and test set (490 complexes), so it can be used to evaluate the generalization ability of the model on complexes. We report the performance of the models on PDBbind-30-nonoverlap in Table 3. The results show that ImagePLB and ImagePLB-P achieve the best performance, except that the Spearman metric of ImagePLB-P is slightly lower than that of ProtMD-P, indicating that ImagePLB and ImagePLB-P have strong generalization capabilities on protein–ligand complex. For more baseline comparisons, such as multimodal (Kyro *et al.* 2023, Zhang *et al.* 2023a, Luo *et al.* 2024, Zhong *et al.* 2024, Kyro *et al.* 2025) or image methods (Zeng *et al.* 2022, Xiang *et al.* 2023, 2024a), see Appendix I, available as supplementary data at *Bioinformatics* online.

**Table 3.** The RMSE, Pearson, Spearman of different methods on PDBBind-30-nonoverlap.^a^

Model	Sequence identity (30%) with nonoverlapping protein–ligand complexes		
	RMSE ↓	Pearson ↑	Spearman ↑
ProtMD	1.411 ± 0.047	0.554 ± 0.024	0.574 ± 0.025
LEFTNet	1.406 ± 0.012	0.567 ± 0.016	0.551 ± 0.021
ImagePLB	**1.402 ± 0.011**	**0.586 ± 0.016**	**0.582 ± 0.020**
GeoSSL-DDM	1.493 ± 0.013	0.555 ± 0.002	0.554 ± 0.005
ProtMD-P	1.371 ± 0.020	0.567 ± 0.016	**0.591 ± 0.013**
Uni-Mol	1.586 ± 0.013	0.452 ± 0.024	0.450 ± 0.020
ImagePLB-P	**1.350 ± 0.023**	**0.598 ± 0.020**	0.590 ± 0.024

a The bold values indicate the best results. Before ImagePLB, there is a comparison of methods without pre-training on molecules and proteins, and after ImagePLB, there is a comparison of pre-training methods. These results are reported with three random seeds.

### 3.4 Ablation study and discussion

#### 3.4.1 Contribution of ligand images

To verify the contribution of ligand images to affinity interactions, we only use LRL and PRL to extract ligand features and protein features, respectively, and splice them together for prediction. For comparison, we replace the ligand image in LRL with the geometric graph of the ligand and encode it using the same EGNN as the 3D pocket encoder. Table 4 shows that when the ligand graph is replaced with an image, the average performance improvement on PDBbind-30 and PDBbind-60 is 3.6% on RMSE, 5.0% on Pearson, and 4.7% on Spearman, which indicates that the image will effectively improve the model’s ability to capture ligand–protein interactions.

**Table 4.** The contribution of ligand images.^a^

Input		Sequence identity (30%)			Sequence identity (60%)		
Ligand	Protein	RMSE ↓	Pearson ↑	Spearman ↑	RMSE ↓	Pearson ↑	Spearman ↑
Graph	Graph	1.517 ± 0.154	0.532 ± 0.072	0.521 ± 0.076	1.472 ± 0.043	0.718 ± 0.016	**0.726 ± 0.019**
Image	Graph	**1.435 ± 0.073**	**0.577 ± 0.029**	**0.574 ± 0.029**	**1.446 ± 0.006**	**0.729 ± 0.000**	0.720 ± 0.004

a The bold values indicate the best results. We replaced the ligand graphs with ligand images under same experimental setup.

#### 3.4.2 Contribution of pre-training

Tables 1 and 2 show that ImagePLB-P outperforms ImagePLB on almost all datasets with up to 9.1% improvement on PDBbind and up to 1.6% improvement on LEP, which proves the effectiveness of the proposed pre-training strategy. For ablation results of the MLNTP pre-training task, see Appendix J.1, available as supplementary data at *Bioinformatics* online.

#### 3.4.3 Contribution of trajectory regularization

**Figure 4.**
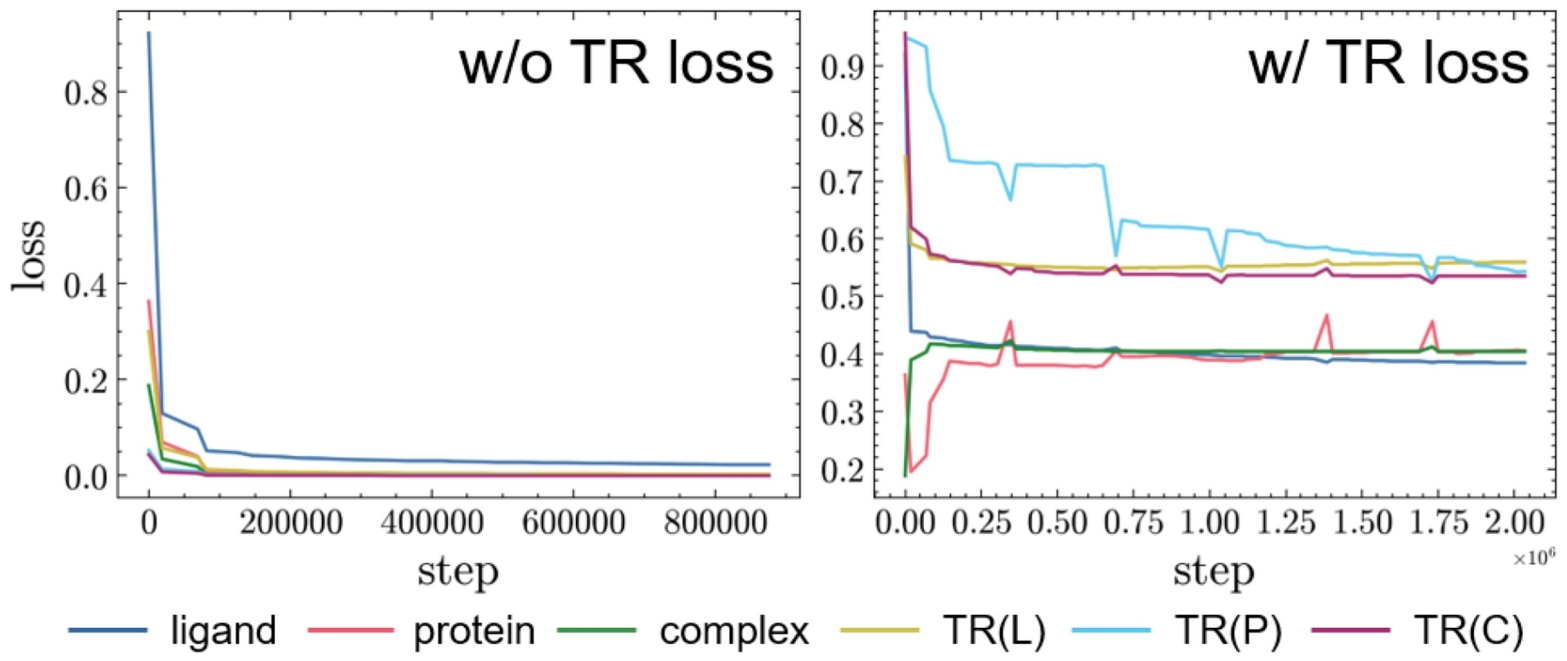
The pre-training loss of MLNTP without and with TR. The *x*-axis and *y*-axis represent the training step and the loss value, respectively. The left figure only shows the loss value without TR. ligand, protein, and complex represent $L_{\mathrm{LNTP}}$, $L_{\mathrm{PNTP}}$, and $L_{\mathrm{CNTP}}$, respectively. TR(L), TR(P), and TR(C) represent $L_{TR}^{L}$, $L_{TR}^{P}$, and $L_{TR}^{C}$, respectively.

**Figure 5.**
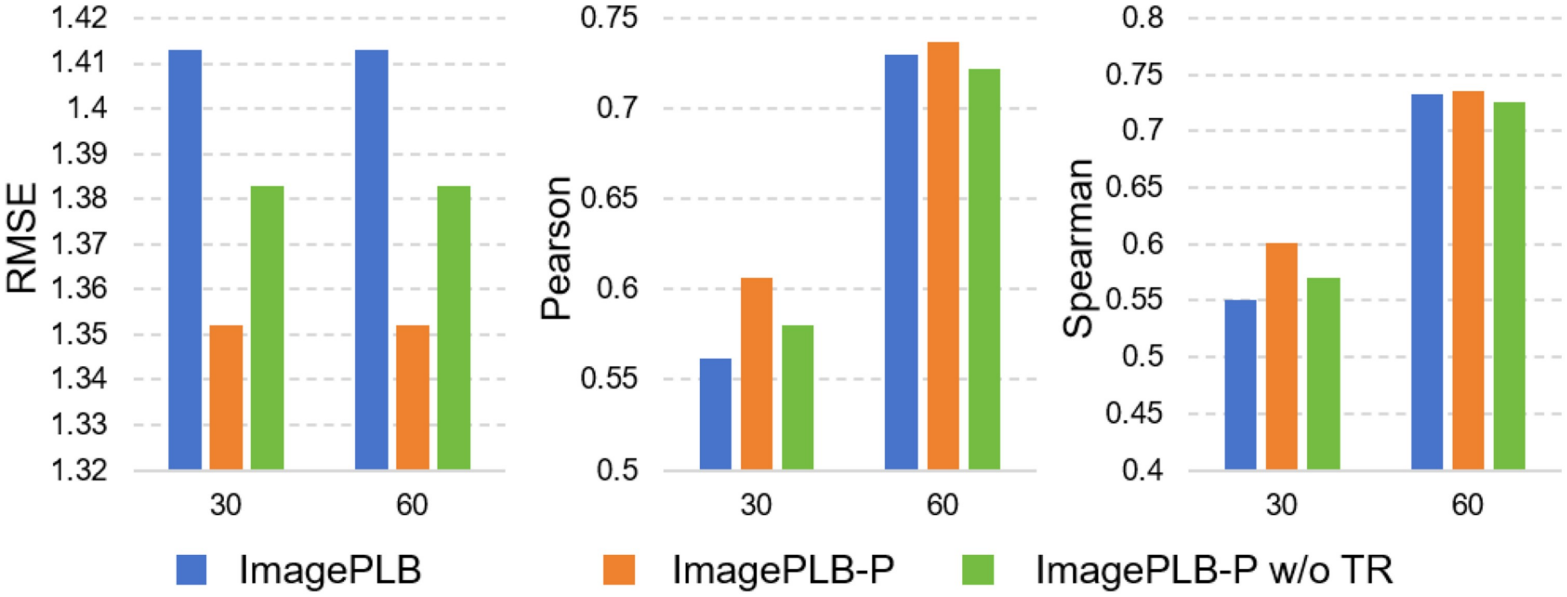
Comparison of ImagePLB, ImagePLB-P, and ImagePLB-P w/o TR in RMSE, Pearson, and Spearman. 30 and 60 represent the PDBbind-30 and PDBbind-60 datasets.

We first compare the pre-training loss with and without trajectory regularization. As shown in Fig. 4, we find that $L_{\mathrm{LNTP}}$, $L_{\mathrm{PNTP}}$, and $L_{\mathrm{CNTP}}$ decrease rapidly when TR is not introduced. However, $L_{TR}^{L}$, $L_{TR}^{P}$, and $L_{TR}^{C}$ are almost 0, indicating that ImagePLB does not learn trajectory-related information but tends to predict adjacent trajectories as almost identical representations. After introducing TR, we find that ImagePLB can learn the differences between adjacent trajectories and has the ability to predict the trajectory of the next frame. In addition, Fig. 5 shows that the performance of ImagePLB-P w/o TR is worse than that of ImagePLB-P, and is even slightly lower than ImagePLB in Spearson and Spearman metrics on PDBbind-60, which demonstrates the advantage of trajectory regularization and can effectively prevent the problem of model collapse.

Moreover, we also discuss the ablation study of ImagePLB on the number of viewpoints (Appendix J.2, available as supplementary data at *Bioinformatics* online), the number of atoms (Appendix J.3, available as supplementary data at *Bioinformatics* online), and the advantage of 3D image compared rdkit-based 2D image (Appendix J.4, available as supplementary data at *Bioinformatics* online), as well as the memory efficiency (Appendix K, available as supplementary data at *Bioinformatics* online) and time complexity (Appendix L, available as supplementary data at *Bioinformatics* online).

## 4 Conclusion

In this work, we first identify two challenges of graph-based PLB, namely the limited ability to capture high-quality PLB representations and the sensitivity to the maximum number of atoms. Then, in order to alleviate these challenges, we propose a novel image-based PLB representation learning framework (called ImagePLB), which is the first attempt to use images to improve the PLB representation. Equipped with multi-level flexible dynamics trajectory pre-training and trajectory regularization, ImagePLB is further pre-trained to perceive the dynamic trajectory changes of ligands and proteins, called ImagePLB-P. Extensive experiments demonstrate that ImagePLB and ImagePLB-P achieve better or competitive performance compared with the existing state-of-the-art methods on PDBbind and LEP. In the future, we will further apply ImagePLB to molecules with more atoms and promote the research and development of macromolecules (e.g. polypeptides or proteins).

## Supplementary Material

btaf535_Supplementary_Data

## Data Availability

All data and implementation details of code can be obtained from figshare (doi: 10.6084/m9.figshare.29209238.v1) and github (https://github.com/HongxinXiang/ImagePLB).
