# Supplementary material for: An image-based protein–ligand binding representation learning framework via multi-level flexible dynamics trajectory pre-training
Source: Bioinformatics. 2025 Sep 24;41(10):btaf535. doi: 10.1093/bioinformatics/btaf535 (PMC12502907; doi:10.1093/bioinformatics/btaf535)
Supplement: btaf535_Supplementary_Data [file btaf535_supplementary_data.pdf]

Supplementary Information: An Image-based Protein-Ligand Binding Representation Learning Framework via Multi-Level Flexible Dynamics Trajectory Pre-training

Hongxin Xiang[1] , Mingquan Liu[1] , Linlin Hou[1] , Shuting Jin[2] , Jianmin Wang[3] , Jun Xia[4] , Wenjie Du[5] , Sisi Yuan[6] , Xiangzheng Fu[7] , Xinyu Yang[1] , Li Zeng[8] , Lei Xu[9,*]

[1]College of Computer Science and Electronic Engineering, Hunan University, Changsha, 410082, Hunan, China

[2]School of Computer Science & Technology, Wuhan University of Science and Technology, Wuhan, 430081, Hubei, China

[3]The Interdisciplinary Graduate Program in Integrative Biotechnology, Yonsei University, Incheon, 03722, Korea

[4]School of Engineering, Westlake University, Hangzhou, 310024, Zhejiang, China

[5]School of Software Engineering, University of Science and Technology of China, Hefei, 230026, Anhui, China

[6]Department of Bioinformatics and Genomics, University of North Carolina at Charlotte, Charlotte, 28223, NC, USA

[7]School of Chinese Medicine, Hong Kong Baptist University, Hong Kong, 999077, SAR, China

[8]Department of AIDD, Shanghai Yuyao Biotechnology Co., Shanghai, 200000, China

[9]School of Electronic and Communication Engineering, Shenzhen Polytechnic University, Shenzhen, 518055, China

Correspondding author: csleixu@szpu.edu.cn

A Related Works

A.1 Protein-Ligand Binding (PLB) Representation Learning

As the available binding data of ligands and proteins continue to increase and their modalities (including sequence, surface and graph) become richer and richer, the field of PLB representation learning has developed rapidly. Recent methods have focused on four aspects (See Appendix A.3 for details of representative methods):

Sequence-based methods. Sequence-based methods [1, 2, 3, 4] represent ligands and proteins as one-dimensional atom and amino acid sequences, and use techniques related to natural language processing (NLP) [5, 6] to develop new methods. For example, DeepDTA [1] accepts the SMILES sequence of the ligand and the amino acid sequence of the protein as input and uses two independent CNN blocks to extract features. DeepAffinity [2] further improves the CNN blocks in DeepDTA to RNN-CNN blocks to capture richer sequence features. CAPLA [3] employs the cross-attention mechanism to capture the mutual effect of protein-binding pocket and ligand. However, these methods do not consider the three-dimensional conformational information of protein and ligand binding.

Surface-based methods. Surfaces are high-level representations of protein structures that display patterns of chemical and geometric features that can fingerprint the interaction patterns of proteins with other biomolecules [7, 8, 9, 10]. MaSIF (Molecular Surface Interaction Fingerprinting) [9] is proposed to capture fingerprints that are important for specific biomolecular interactions, improving performance across binding pocket and protein interface prediction tasks. HoloProt [11] introduces a multi-scale graph construction method, which connects surface to structure and sequence and shows a performance improvement on ligand binding affinity. MFE [10] incorporates information from protein surfaces, 3D structures, and sequences. However, these methods require the additional surface information, resulting in high computational complexity, especially in large protein systems [12].

Voxel-based methods. Voxels, also known as 3D pixels, are 3D cubes located on a 3D grid used to create 3D models, which are widely studied in computer vision, such as medical imaging [13, 14], 3D object detection [15], and autonomous driving [16]. 3DCNN [17] represents ligands and proteins as fixed-size cubes in 3D space, which are discretized into voxels with a resolution of 1 Å to form a grid, and uses 4 layers of 3D-convolutional layers to extract features from the voxels. However, due to the very high dimensionality of voxels, the model training process becomes very complex and slow, leading to unpopular applications.

Graph-based methods. Given that atoms and amino acids in ligands and proteins can be directly represented as nodes in a graph, graph-based methods have become very popular [18, 19, 20, 21, 22, 23]. GraphDTA [18] represents ligands as a two-dimensional (2D) graph structure and learns the binding representation between the graph and the amino acid sequence. However, the 2D graph structure cannot truly reflect the geometric conformation of the ligand-protein complex. Therefore, many methods construct the three-dimensional (3D) geometric conformation of the ligand and protein into a 3D graph and learn the binding representation between the ligand and the protein, such as GeoSSL-DDM [19], ProtMD [20], DrugCLIP [22].

Different from all previous methods, ImagePLB is the first image-based PLB representation learning framework, which considers cross-modal 3D conformational interaction between ligand images and protein graphs.

A.2 Pre-training Strategy in PLB

With the rise of self-supervised learning, more and more pre-training strategies have been proposed to enhance the generalization of models on PLB representation learning. SSM-DTA [4] uses two 12-layer $\text{RoBERTa}_{\text{base}}$ [24] and semi-supervised training strategy to pre-train on large-scale unlabeled molecule and protein data. GeoSSL-DDM [19] proposes a denoising distance matching strategy to simulate the continuous motion of molecules in three-dimensional Euclidean space to form a smooth potential energy surface, which effectively improves the ability to extract basic features from geometric structures. DrugCLIP [22] explicitly aligns representations between ligands and 3D pockets by leveraging contrastive learning [25] on PDBbind [26], BioLip [27], and ChEMBL [28]. Considering that the binding of ligand and protein is a dynamic process, ProtMD [20] proposes 2 pre-training tasks (prompt-based denoising generative task and snapshot ordering task) on 63 protein-ligand complexes with 100ns dynamic trajectories and demonstrates that there is a strong correlation between the amplitude of conformational movement in three-dimensional space and the strength of binding of the ligand to its receptor. Different from ProtMD, we find that simply making predictions between adjacent conformations can lead to the model collapse due to the over-similarity of features between adjacent trajectories. Furthermore, we propose a novel multi-level next trajectory prediction pre-training strategy with trajectory regularization and pre-train ImagePLB on the flexible dynamics trajectories of 16,972 protein-ligand complexes.

A.3 Representative Methods in Protein-Ligand Binding (PLB) Representation Learning

We summarize the existing representative methods in Table S2. According to the different representations, these methods are divided into 4 categories:

- **Sequence-based methods:** DeepDTA, DeepAffinity.
- **Surface-based methods:** MaSIF, HoloProt.
- **Voxel-based methods:** 3DCNN.
- **Graph-based methods:** GraphDTA, GeoSSL-DDM, ProtMD, LEFTNet, DrugCLIP, Uni-Mol.

ImagePLB extends the existing PLB representation learning methods, which is a pioneer work to explore image-based PLB representation methods.

B Motivation Analysis

Table S1: 4 different model architectures to verify our motivations.

Name	Ligand Encoder	Protein Encoder	Ligand Data	Protein Data
EE	EGNN	EGNN	3D graph	3D graph
RE	ResNet18	EGNN	Image	3D graph
SS	SE(3) Transformer	SE(3) Transformer	3D graph	3D graph
RS	ResNet18	SE(3) Transformer	Image	3D graph

Here, we describe the two main motivations for ImagePLB in details. We first points the details of our experiments. In general, we use a ligand encoder and a protein encoder to extract the features of the ligand and protein respectively and concatenate these features into a linear layer to predict affinity. For protein, we use the protein region within 6 Å near the ligand as a pocket and input it into the protein encoder to extract features. As shown in Table S1, we set up 4 different model architectures to verify our motivations:

- EE: 2 separate EGNNs for ligand encoder and protein encoder respectively;
- RE: a ResNet18 for ligand encoder and an EGNN for protein encoder;
- SS: 2 separate SE(3) Transformers for ligand encoder and protein encoder respectively;
- RS: a ResNet18 for ligand encoder and an SE(3) Transformer for protein encoder.

We use the exact same experimental settings, i.e., train on PDBbind-30 for 1,000 epochs with a batch size of 16, a learning rate of 5e-5, and a dropout of 0.15. For EGNN and SE(3) Transformer, in order to study whether the maximum number of atoms in the ligand affects their performance, we also try to train them under different maximum number of atoms.

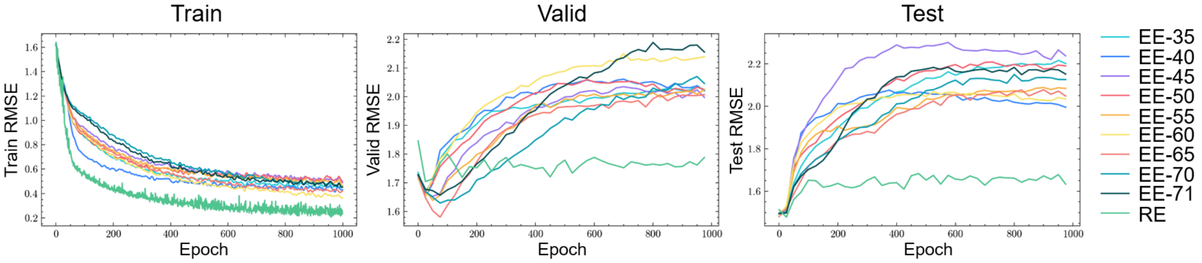

Figure S1: RMSE curves of EE and RE on the training set, test set and validation set on PDBbind-30 for 1,000 epochs. The number after EE indicates the maximum number of atoms in the ligand supported by the ligand encoder. For example, EE-35 means that the maximum number of atoms in the ligand is 35. If it exceeds 35, the extra atoms will be truncated.

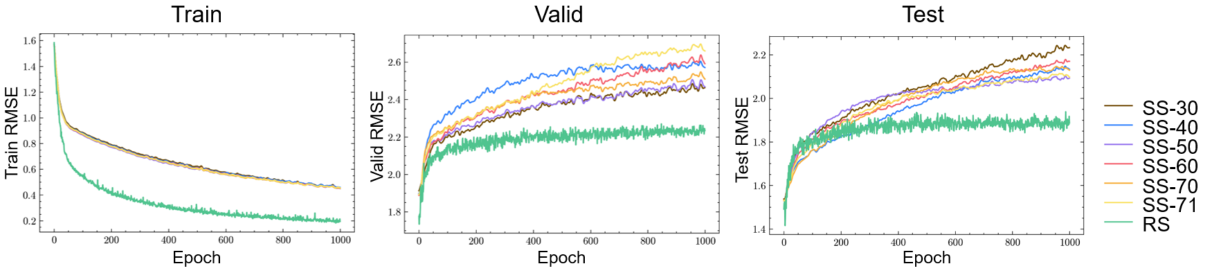

Figure S2: RMSE curves of SS and RS on the training set, test set and validation set on PDBbind-30 for 1,000 epochs. The number after SS indicates the maximum number of atoms in the ligand supported by the ligand encoder.

- **Being sensitive to the maximum atomic length.** As shown in Figure S1 and Figure S2, we can clearly see that EE and SS are sensitive to the maximum number of atoms in the ligand. In particular, they have larger performance variance on the validation set and the test set. However, for RE and RS, the ligand encoder used is atom number-independent, which uses images to represent ligands, making it able to perfectly overcome this problem regardless of the atom number.

Table S2: Representative methods in PLB representation learning, including non-pretrained and pretrained methods.

Methods	Pretrained?	Input modality		encoder	
		Ligand	Protein	Ligand	pocket
DeepDTA [1]		Sequence (SMILES)	Sequence (amino acid)	CNN blocks	CNN blocks
GraphDTA [18]		Graph (2D)	Sequence (amino acid)	GCN/GAT/GIN/GAT-GCN	CNN blocks
DeepAffinity [2]		Sequence (SMILES)	Sequence (amino acid)	RNN-CNN blocks	RNN-CNN blocks
MaSIF [9]	N	Graph (3D)	Surface (3D, pocket)	MPN	CNN
3DCNN [17]		Voxel (3D)	Voxel (3D, pocket)	CNN	
HoloProt [11]		Graph (3D)	Graph (3D, surface+structure)	MPN	MPN
LEFTNet [21]		Graph (3D)	Graph (3D, pocket)	LEFTNet	
SSM-DTA [4]		Sequence (SMILES)	Sequence (amino acid)	$\text{RoBERTa}_{\text{base}}$	$\text{RoBERTa}_{\text{base}}$
ProtMD [20]		Graph (3D)	Graph (3D, pocket)	EGNN	
GeoSSL-DDM [19]	Y	Graph (3D)	Graph (3D, pocket)	PaiNN	
Uni-Mol [23]		Graph (3D)	Graph (3D, pocket)	SE(3) Transformer	SE(3) Transformer
DrugCLIP [22]		Graph (3D)	Graph (3D, pocket)	SE(3) Transformer	SE(3) Transformer
ImagePLB (Ours)	N	Image	Graph (3D, pocket)	ResNet18	EGNN
ImagePLB-P (Ours)	Y	Image	Graph (3D, pocket)	ResNet18	EGNN

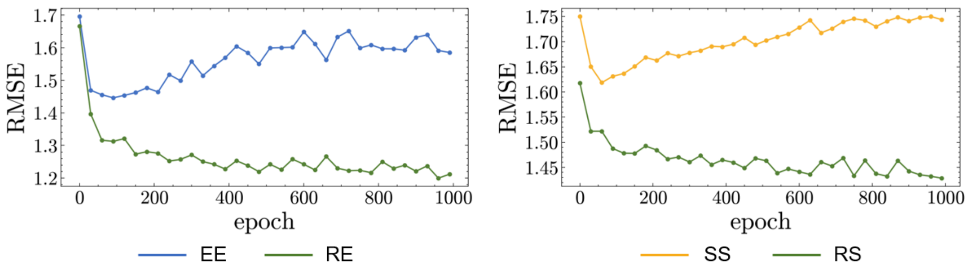

Figure S3: The average RMSE performance curves of EE, RE, SS, and RS in PDBbind-30 for 1,000 epochs, which are calculated by averaging the RMSE of the training set, validation set, and test set. The average RMSE performance reflects the comprehensive learning ability of the model, including fitting ability and generalization ability. The maximum number of atoms for EE and SS is set to 71 because the maximum number of atoms on PDBbind-30 is 71.

- **Limited in capturing high-quality protein-ligand binding (PLB) representations.** From Figure S1 and Figure S2, we find that RE and RS converge faster than EE and SS in training and achieve stable performance on the validation set and test set, which means that RE and RS can learn stable high-quality PLB representations. In contrast, EE and RE are very easy to overfit. Further, to better demonstrate the advantages of RE and RS on PLB, we calculated the average performance of RE and RS on the training set, validation set and test set. As shown in Figure S3, we find that RE and RS can continuously decrease to obtain lower RMSE performance. However, EE and SS can no longer learn more information related to PLB after about 75 epochs.

Overall, these experiments confirm the advantages of the image-based PLB model and motivate us to develop ImagePLB for more accurate ligand-protein binding predictions.

C Details of Multi-View Ligand Images

Here, we provide a detailed description of the multi-view ligand image generation process and summarize the corresponding PyMOL commands in Algorithm 1:

- **#1:** Load the 3D conformation file of the ligand into PyMOL using the command `load` $file_{conf}$. The input file contains the geometric information of the molecule and can be in formats such as .sdf, .xyz, or .mol.
- **#2:** To improve visual clarity, we set the background color of the image to white using `bg_color white`.
- **#3:** Visualize the molecule in a ball-and-stick style using `set stick_ball, on; set stick_ball_ratio, 3.5; set stick_radius, 0.15; set sphere_scale, 0.2`. Specifically, `set stick_ball, on` enables ball-and-stick rendering; `set stick_ball_ratio, 3.5` controls the relative size of the spheres (atoms) to the sticks (bonds); `set stick_radius, 0.15` sets the thickness of the sticks (in Å); `set sphere_scale, 0.2` adjusts the scale of the atom spheres.
- **#4:** Display valence information using `set valence, 1; set valence_mode, 0; set valence_size, 0.1`. In detail, `set valence, 1` enables visualization of bond multiplicity (e.g., double or triple bonds); `set valence_mode, 0` controls the layout of valence lines. `set valence_size, 0.1` defines the thickness of valence lines.
- **#5:** Generate different viewing angles by rotating the molecule using `rotate` $axis$, $angle$.
- **#6:** Save the rendered molecular image to a specific path using `save` $path$.

To further illustrate the rendering process, we provide step-by-step visual examples corresponding to each stage in Figure S4.

D PDBbind and data splits

The database version used by PDBbind-30, PDBbind-60, and PDBbind-scaffold is PDBbind v2019 [29]. The details of the splits are as follows:

- PDBbind-30: A non-overlapping split based on 30% protein sequence identity, where no protein in the test set shares more than 30% sequence identity with any protein in the training set. This setting is intended to assess generalization to novel protein families.
- PDBbind-60: A less strict version using a 60% sequence identity threshold, allowing moderate homology between training and test proteins.

Algorithm 1 The main PyMol command of the generation of multi-view ligand images.

```
Input: The save path path, the axis axis and angle angle of view rotation
for sampled a conformer file of the ligand file_conf from files_conf do
    #1 Load the ligand image from the conformer file
    load file_conf
    #2 Set the background color of the ligand image
    bg_color white
    #3 Set the ligand image to a ball-and-stick model
    set stick_ball,on;set stick_ball_ratio,3.5;set stick_radius,0.15;set sphere_scale,0.2
    #4 Set the display mode of molecular valence bonds
    set valence,1;set valence_mode,0;set valence_size,0.1
    #5 Rotate the image by angle degrees along the axis axis
    rotate axis, angle
    #6 Save the ligand image to path
    save path
end for
```

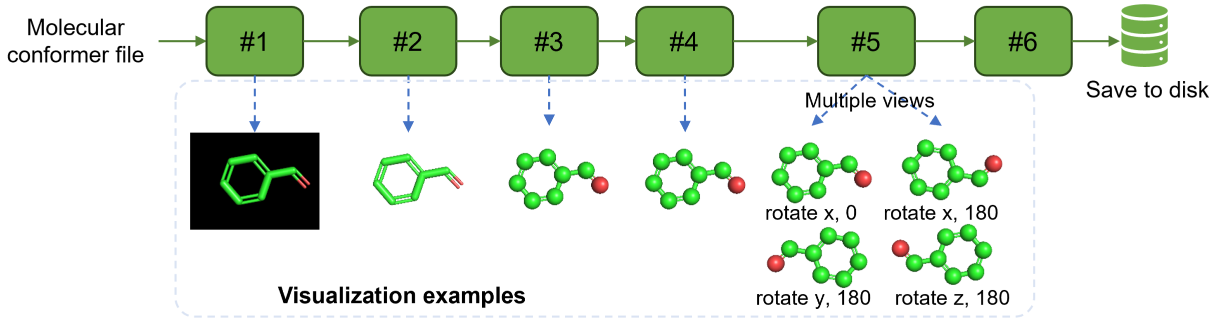

Figure S4: The generation process of multi-view ligand images using PyMol.

- PDBbind-scaffold: A ligand-based scaffold split based on Bemis–Murcko scaffolds, where molecules with the same core scaffold are grouped into the same subset. This split is designed to measure the model's ability to generalize to chemically novel compounds.

E Baselines

In order to comprehensively evaluate ImagePLB, we selected four categories of methods with different representation types (sequence, surface, voxel and graph) as baselines for comparison, including methods that are not pre-trained on molecules and methods that are pre-trained on molecules. All baselines are summarized in Table S3. In evaluation of PDBbind-30, PDBbind-60, and PDBbind-Scaffold, ProtMD and ProtMD-P represent the results without pre-training and pre-training respectively. The results of SSM-DTA, LEFTNet, Uni-Mol, DrugCLIP on 30%, 60%, and Scaffold and ProtMD, ProtMD-P on 60% and Scaffold are reproduced by us using publicly available code and other results are obtained from HoloProt [11]. In evaluation of LEP, the results of GeoSSL-DDM are from [19], SchNet, LeftNet, PaiNN, Equiformer are reproduced by us using publicly available code and other results are from ProtMD-P [20].

F Datasets

Pre-training dataset. In pre-training stage, we use MISATO [30] as the original pre-training data. MISATO consists of molecular dynamics simulations of about 20,000 experimental protein-ligand complexes, which starts from existing experimental structures and systematically optimizes these structures using semi-empirical quantum mechanics, including a large number of molecular dynamics trajectories of explicit protein-ligand complexes in water, accumulating more than 170μs.

Here, we describe in detail the process of using dynamics simulation (MD) to generate trajectories, which will be used for pre-training of ImagePLB. All MD simulations were performed using the Amber20 software suite. Ligands were parameterized using the GAFF2 force field, and AM1-BCC charges were assigned via Antechamber. In cases where charge calculation failed to converge within 1 hour, AM1 charges computed using ULYSSES were employed. Protein force fields were modeled using ff14SB. Each complex was neutralized with Na^+ and Cl^- ions and solvated in an octahedral TIP3P water box with periodic boundary conditions and a minimum 12 Å buffer around the solute. Energy minimization (1,000 steps steepest descent followed by conjugate gradient) and gradual heating to 300 K over 16 ps were performed. Finally, production MD simulations were run in

Table S3: Summary of the current state-of-the-art baselines.

**Model**	Category	pretrained dataset
DeepDTA	sequence-based	-
SSA	sequence-based	-
TAPE	sequence-based	-
ProtTrans	sequence-based	-
MaSIF	surface-based	-
HoloProt	surface-based	-
3DCNN	voxel-based	-
IEConv	3D graph-based	-
ProtMD w/o pretrain	3D graph-based	-
LEFTNet	3D graph-based	-
ImagePLB	image-based	-
SSM-DTA	sequence-based	10 million molecules from PubChem and 10 million proteins from Pfam
GeoSSL-DDM	3D graph-based	PubChemQC (4 million molecules with 3D geometrics)
ProtMD-P	3D graph-based	63 protein-ligand complexes with 100ns dynamic trajectories
Uni-Mol	3D graph-based	209M molecular 3D conformations + 3M candidate protein pocket data
DrugCLIP	3D graph-based	PDBbind + BioLip (122,861 protein-molecule pairs) + ChEMBL
ImagePLB-P	image-based	Dynamic trajectories of 16,972 complexes with 10ns

Table S4: The statistical information of downstream tasks. #Sample represents the total number of complexes. #Train, #Valid, #Test represent the number of complexes in the training set, validation set, and test set, respectively.

**Datasets**	**#Sample**	**#Train**	**#Valid**	**#Test**	**Task Type**
PDBbind-30	4,463	3,507	466	490	Regression
PDBbind-60	4,463	3,563	448	452	
PDBbind-Scaffold	4,709	3,767	471	471	
LEP	512	300	110	102	Classification

the NVT ensemble for 10 ns. After acquiring 10 ns of trajectory data, the first 2 ns were discarded as the equilibration phase, and the remaining 8 ns were stored for analysis, consisting of 100 snapshots per complex. We use these trajectory snapshots for pre-training of ImagePLB.

As shown in Figure S5, the number of atoms in the ligand and the corresponding pocket in the pre-training dataset is shown. We decompose the complex in MISATO into separate proteins and ligands. Then, we search for pockets within 8Å of the protein based on the geometric information of the ligand. Note that if we do not find a pocket within 8Å, we will gradually increase the distance by 1Å until the pocket is found. Finally, we use OpenBabel [31] to convert the ligand data into sdf format and use PyMol [32] to render multi-view molecular images. Considering rendering efficiency, we only render multi-view images for the first frame of the dynamics simulation and use the original image perspectives for other frames, which has been experimentally verified to have little significant effect difference.

Downstream datasets. The Table S4 shows the statistical information of downstream tasks, including PDBbind-30, PDBbind-60, and PDBbind-Scaffold. We search for pockets within 8Å of the protein based on the geometric information of the ligand. Furthermore, we also count the distribution of the number of atoms in the training set, as shown in Figure S6. Please note that we did not compare the results of HoloProt on PDBbind-60 since we used the latest data from Atom3D, which has 3563 training sets, 448 validation sets, and 452 test sets while the data used by HoloProt has 3,678 training sets, 460 validation sets, and 460 test sets.

G Computational Cost of the Pretraining

In our current implementation, the pretraining of ImagePLB takes approximately 9 days running on 4 NVIDIA GeForce RTX 4090 GPUs. Although the computational cost is substantial, we believe it is justified by the consistent and significant performance gains demonstrated across multiple downstream tasks. Furthermore, the pretrained model serves as a valuable reusable asset that can be fine-tuned or adapted for diverse applications, thereby amortizing the initial investment and benefiting the broader research community.

To improve scalability, several strategies can be considered in future work, such as optimizing MD simulation protocols with accelerated sampling techniques, employing more efficient 3D data representations (e.g., sparse convolutions or voxel compression), or exploring hybrid approaches that combine dynamic graph and image-based features. These directions hold promise for reducing computational demands while maintaining competitive performance.

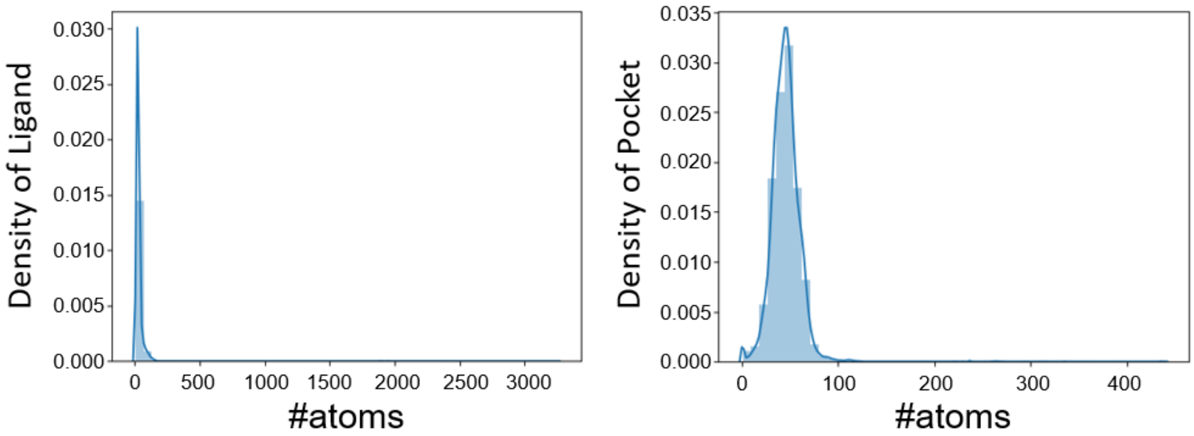

Figure S5: The distribution of the number of atoms in the training set from the pre-training dataset (MISATO). #atoms represents the number of atoms. Note that atoms in pockets refer to α-C atoms.

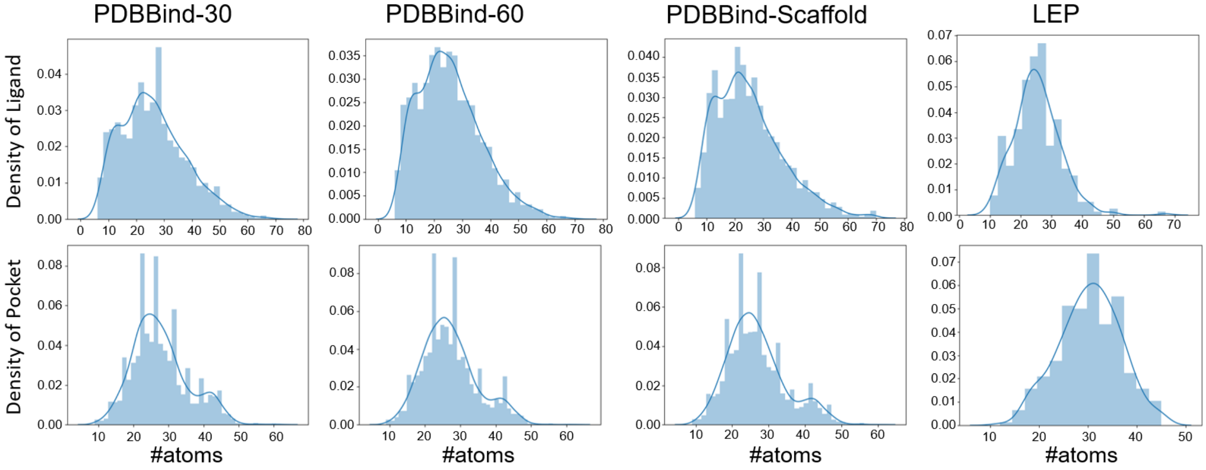

Figure S6: The distribution of the number of atoms in the training set for the downstream task dataset. #atoms represents the number of atoms. Note that atoms in pockets refer to α-C atoms.

H Hyperparameter Optimization

The hyperparameter search space is shown in Table S5, which is determined based on our experience. We compute the mean and variance of the performance by performing 3 separate runs under the same hyperparameter combination with 3 different random seeds (0, 1, 2).

I Experimental Comparison with More Baselines

I.1 Comparison with HAC-Net and T-ALPHA

We compare ImagePLB with two recent multimodal state-of-the-art methods: HAC-Net [33] and T-ALPHA [34].

HAC-Net represents the protein–ligand complex using both a *voxel-based representation* and a *graph-based representation*, employing a 3D convolutional neural network (3D-CNN) for voxel features and two graph convolutional networks (GCNs) for the protein and ligand graphs, respectively. T-ALPHA adopts a more comprehensive multimodal design and separately encodes the protein, the ligand, and the protein–ligand complex:

- For the protein, it constructs:
  1. A *protein pocket graph*, processed by an E(n) Equivariant GNN,
  2. A *protein pocket surface point cloud*, processed using quasi-geodesic convolution layers,
  3. A *protein amino acid sequence embedding*, extracted via the ESM-2 language model.
- For the ligand, it uses:

Table S5: The hyperparameter search space on PDBbind-30, PDBbind-60, PDBbind-Scaffold and LEP datasets.

	**30/scaffold split**	**60 split**	**LEP**
epoch	[10, 15, 30]	[40]	[100]
lr	[1e-4, 3e-4, 5e-4, 8e-4]		[8e-5, 1e-4, 3e-4]
batch	[16, 32]		[16]
pocket/predictor dropout	[0.15, 0.3, 0.5]		

1. A *ligand graph*, processed with an E(n) Equivariant GNN,
2. A *209-dimensional fingerprint descriptor*,
3. A *SMILES-based embedding*, generated by a SMILES-pretrained transformer.

- The protein–ligand complex is represented as a *unified graph*, processed by an E(n) Equivariant GNN.

ImagePLB, in contrast, introduces a fundamentally different approach. It constructs *MD-driven 3D image-based representations of the ligand*, which capture the *dynamic and time-dependent interactions* with the protein. This representation allows ImagePLB to model flexible binding behaviors that static representations—such as graphs, voxels, point clouds, or sequences—as used in HAC-Net and T-ALPHA, cannot effectively capture.

To further support this difference, we conduct additional experiments on the PDBbind-30 dataset, a challenging benchmark with strong distribution shift, simulating real-world scenarios. As shown in Table S6, ImagePLB-P achieves the best performance among the three models, demonstrating superior generalization and effectiveness.

Table S6: Performance comparison of ImagePLB-P, HAC-Net, and T-ALPHA on the PDBbind-30 dataset.

**Model**	**Sequence Identity (30 %)**		
	RMSE	Pearson	Spearman
HAC-Net	1.413±0.036	0.600±0.004	0.589±0.000
T-ALPHA	1.688±0.307	0.503 ±0.012	0.485 ±0.016
ImagePLB-P	**1.352±0.021**	**0.606±0.012**	**0.601±0.016**

I.2 Comparison with Image-based Metohds

To strengthen the comparative evaluation, we selected four recent image-based pretraining methods as baselines: ImageMol [35], CGIP [36], IEM-2D, and IEM-3D [37]. The results are summarized in Table S7. We observe that ImagePLB-P consistently outperforms all other image-based approaches, demonstrating the effectiveness and superiority of the proposed method.

Table S7: Comparison with image-based methods on the PDBbind-30 and PDBbind-60 datasets.

**Model**	**Sequence Identity (30 %)**			**Sequence Identity (60 %)**		
	RMSE	Pearson	Spearman	RMSE	Pearson	Spearman
ImageMol	1.423±0.030	0.584±0.027	0.578±0.027	1.446±0.025	0.711±0.013	0.713±0.014
CGIP	1.400±0.015	0.590±0.012	0.579±0.011	1.394±0.009	0.734±0.004	0.729±0.004
IEM-2D	1.451±0.057	0.558±0.015	0.546±0.022	1.452±0.020	0.708±0.005	0.706±0.004
IEM-3D	1.397±0.040	0.581±0.023	0.570±0.017	1.574±0.016	0.469±0.007	0.470±0.007
ImagePLB-P	**1.352±0.021**	**0.606±0.012**	**0.601±0.016**	**1.379±0.008**	**0.737±0.004**	**0.735±0.005**

I.3 Comparison with Multi-Modality Metohds

We further selected three additional multimodal methods as baselines for comparison, namely MMDTA[38], ConBap[39], and FM-DTA [40]. We then evaluated the performance of all baselines on the PDBBind-30 dataset. As shown in Table S8, ImagePLB-P achieves the best performance among all compared methods, demonstrating the effectiveness of the proposed approach.

Table S8: Performance comparison with additional multimodal baselines on PDBbind-30.

**Model**	**Sequence Identity (30 %)**		
	RMSE	Pearson	Spearman
MMDTA	1.567±0.011	0.496±0.005	0.490±0.006
ConBap	1.477±0.023	0.583±0.005	0.575±0.007
FM-DTA	1.682±0.080	0.363±0.033	0.352±0.036
ImagePLB-P	**1.352±0.021**	**0.606±0.012**	**0.601±0.016**

J More Ablation Studies

J.1 Ablation Study about Multi-Level Next Trajectory Prediction (MLNTP) Task

We conducted a detailed investigation into the impact of each level—ligand, protein, and complex—within MLNTP on the performance of ImagePLB. Specifically, we pre-trained ImagePLB using only one of the objectives, $\mathcal{L}_{LNTP}$, $\mathcal{L}_{PNTP}$, or $\mathcal{L}_{CNTP}$, and then evaluated the model on downstream tasks. As shown in Table S9, pre-training with a single level (Ligand only, Protein only, or Complex only) consistently outperformed the model trained without any level-specific pre-training ("None"), demonstrating the effectiveness of each individual level. Notably, combining all three levels for pre-training ("All") achieved the best performance, indicating that the integration of different levels can provide complementary benefits and further enhance the model beyond what is possible with any single level alone.

Table S9: The ablation study of multi-level next trajectory prediction (MLNTP). L, P and C represent ligand level, protein level and complex level, respectively. "Ligand only", "Protein only" and "Complex only" mean using only $\mathcal{L}_{LNTP}$, $\mathcal{L}_{PNTP}$ and $\mathcal{L}_{CNTP}$ losses, respectively.

**Model**	**Level**			**Sequence Identity (30 %)**			**Sequence Identity (60 %)**		
	**L**	**P**	**C**	**RMSE**	**Pearson**	**Spearman**	**RMSE**	**Pearson**	**Spearman**
None	×	×	×	1.413±0.008	0.562±0.015	0.551±0.012	1.396±0.041	0.730±0.018	0.733±0.021
Ligand only	✓	×	×	1.387±0.046	0.578±0.041	0.570±0.048	1.391±0.018	0.734±0.007	0.727±0.006
Protein only	×	✓	×	1.390±0.055	0.596±0.018	0.588±0.013	1.395±0.040	0.735±0.014	0.727±0.008
Complex only	×	×	✓	1.384±0.043	0.585±0.021	0.573±0.027	1.393±0.008	0.732±0.001	0.733±0.003
All	✓	✓	✓	**1.352±0.021**	**0.606±0.012**	**0.601±0.016**	**1.379±0.008**	**0.737±0.004**	**0.735±0.005**

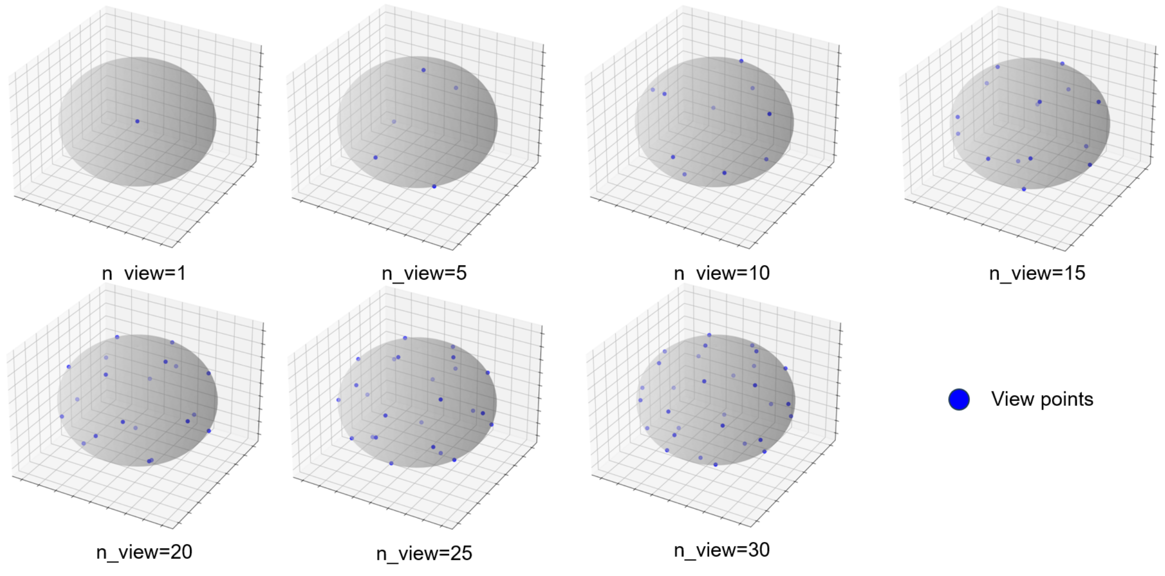

Figure S7: Generated uniform viewpoints. n_view represents the number of views.

Table S10: The effect of the atom number of ligand on PDBbind-30 dataset. #atom represents the atom number of ligand. 5-10 means the range where the number of atoms is greater than or equal to 5 and less than 10.

#atom	RMSE	Spearman	Pearson
5-10	1.417±0.101	0.396±0.103	0.559±0.038
10-15	1.448±0.037	0.565±0.029	0.548±0.036
15-20	1.250±0.039	0.646±0.026	0.656±0.020
20-25	1.347±0.027	0.574±0.012	0.552±0.011
25-30	1.305±0.020	0.655±0.011	0.679±0.013
30-35	1.280±0.010	0.581±0.016	0.605±0.007
35-40	1.427±0.049	0.557±0.070	0.581±0.040
40-45	1.850±0.017	0.660±0.031	0.670±0.008
45-50	1.097±0.082	0.607±0.077	0.695±0.026

Table S11: The effect of the atom number of ligand on LEP dataset. #atom represents the atom number of ligand. 10-15 means the range where the number of atoms is greater than or equal to 10 and less than 15.

#atom	ACC	AUROC	AUPRC
10-15	0.848±0.043	0.967±0.047	0.750±0.354
15-20	0.844±0.083	0.987±0.019	0.973±0.038
20-25	0.697±0.119	0.766±0.084	0.774±0.064
25-30	0.700±0.108	0.764±0.088	0.826±0.075
30-35	0.825±0.025	0.962±0.029	0.922±0.063
40-45	0.889±0.079	0.958±0.059	0.981±0.027

J.2 Ablation Study about The Number of Views

Viewpoint refers to the angle from which we observe the target. We formulate the viewpoint selection as a problem of uniformly sampling points on a sphere and use the Fibonacci sphere sampling algorithm [41] to obtain these points. In detail, assuming we need to generate n viewpoints, we first obtain a golden angle in radians $\phi = \pi \times (\sqrt{5} - 1)$. Then, the i-th viewpoint (x, y, z) on the sphere is sampled according to the following formula:

$$y = 1 - (\frac{i}{n-1}) \times 2 \tag{1}$$

$$x = \cos(\phi \times i) \times \sqrt{1 - y^2} \tag{2}$$

$$z = \sin(\phi \times i) \times \sqrt{1 - y^2} \tag{3}$$

Finally, we convert the coordinates on the sphere into the angle of the rendered image:

$$rotation_x = degree(arctan2(\sqrt{y^2 + z^2}, x)) \tag{4}$$

$$rotation_y = degree(arctan2(\sqrt{x^2 + z^2}, y)) \tag{5}$$

$$rotation_z = degree(arctan2(\sqrt{x^2 + y^2}, z)) \tag{6}$$

where the functions *degree* and *arctan2* come from the numpy library [42]. Here we generate {1, 5, 10, 15, 20, 25, 30 } viewpoints and render multi-view images. The specific viewpoints are shown in Figure S7. We tune the parameters from {60, 100} epochs, {8e-5, 1e-4, 3e-4} learning rates, and {0.15, 0.5} dropout and report the ROC-AUC performance.

As shown in Figure S8, we find that the number of views has an impact on ImagePLB-P performance, which shows that we can improve the performance of the model by adjusting the number of appropriate views. In particular, we find that the ACC of ImagePLB-P has an increasing trend when the number of views increases from 1 to 30 with the performance increasing from 72.6% to 78.4%, and ImagePLB achieves the best ACC (=78.4%) and AUROC (=86.3%), AUPRC (=83.7%) when the number of views is 25 and 30, respectively, suggesting that we may further improve the performance by increasing the number of views.

J.3 Ablation Study on The Effect of The Number of Atom

Here, we study the effect of different atomic numbers on the performance of ImagePLB-P on PDBbind-30 and LEP datasets, and show the results of ImagePLB-P in Figure S9. Figure S9 shows the performance of the ImagePLB-P for ligands with different numbers of atoms in PDBBind-30 and LEP. We find that the performance fluctuates in the range of different atomic numbers,

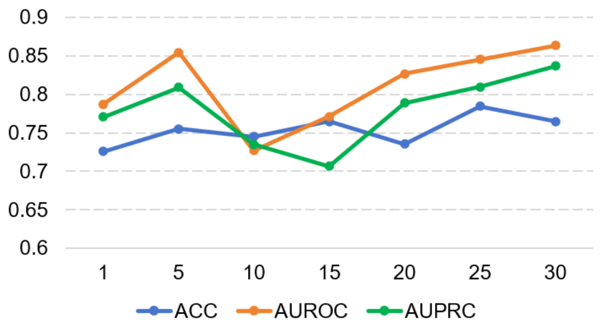

Figure S8: Performance of ImagePLB-P under different numbers of views (1, 5, 10, 15, 20, 25, 30).

suggesting that ImagePLB-P has a preference for molecules with different numbers of atoms. In addition, since no significant correlation is observed between the performance of ImagePLB-P and the number of atoms, the performance of ImagePLB-P may also be related to the distribution of molecular data within each interval of the number of atoms. We also show the details of results in Table S10 and Table S11.

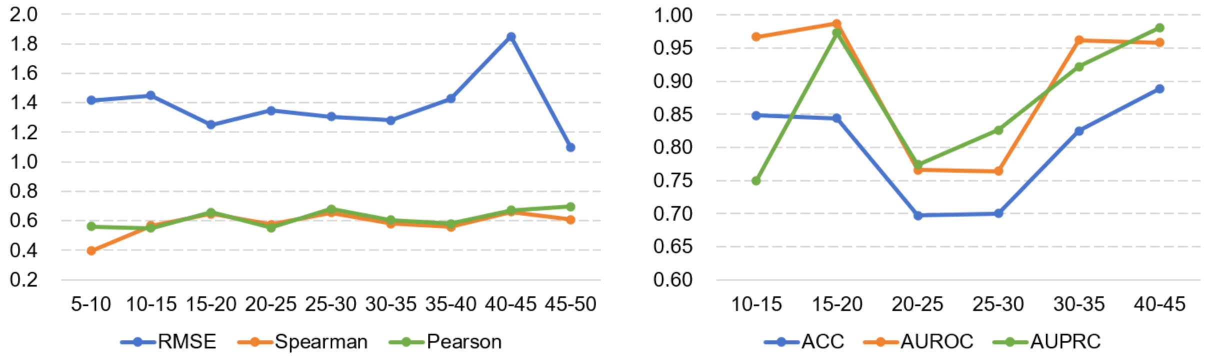

Figure S9: Performance of ImagePLB-P in different ranges of atomic numbers of the ligand. The left and right sub-figures show the results of PDBBind-30 and LEP, respectively.

J.4 The Advantages of 3D Images Compared with 2D Images

To demonstrate the motivation for using 3D ligand images, we investigated the performance advantage of 3D over 2D image representations. Specifically, we replaced the 3D ligand images in ImagePLB with 2D ligand depictions generated by RDKit, and evaluated the resulting variants on the PDBbind-30 and PDBbind-60 benchmarks.

As shown in Table S12, the 3D image-based variant of ImagePLB achieves better overall performance. These results highlight the effectiveness of the multi-view 3D ligand image representation used in ImagePLB.

Table S12: Comparison of 2D and 3D ligand image representations in ImagePLB on PDBbind-30 and PDBbind-60.

**Model**	**Image**	**Sequence Identity (30 %)**			**Sequence Identity (60 %)**		
		RMSE	Pearson	Spearman	RMSE	Pearson	Spearman
ImagePLB	2D	1.421±0.065	**0.565±0.036**	**0.564±0.035**	1.431±0.015	0.714±0.006	0.710±0.008
	3D	**1.413±0.008**	0.562±0.015	0.551±0.012	**1.396±0.041**	**0.730±0.018**	**0.733±0.021**

K Memory Efficiency

We compare the memory consumption during training. Here, we use EGNN and SE(3) Transformer (SE3T for short) as representatives of graph-based PLB frameworks, which uses geometric graphs to represent ligands and proteins. For fair comparison, we only use a single-layer SE3T model with 2 heads, 24 head dimensions, and 60 hidden dimensions. As shown in Table S13, we find that the GPU memory consumption increases with the maximum number of atoms in the ligand. However, the image is not affected by the maximum number of atoms. When the maximum atom length reaches 200, the memory consumption

of ImagePLB (EGNN) can be reduced by 38.3% compared with the EGNN-based PLB method, and when the maximum atom length reaches 90, the memory consumption of ImagePLB (SE3T) can be reduced by 58.2% compared with the SE3T-based PLB method. It is worth noting that the memory efficiency of ImagePLB will further increase when the number of atoms continues to increase. Currently, molecular research is increasingly biased towards larger molecules with more than 200 atoms. We believe that ImagePLB will be more memory-efficient in large-molecule development compared to graph-based and sequence-based PLB methods.

Table S13: Comparison of EGNN-based PLB method, SE3T-based method and ImagePLB on memory efficiency. The maximum atoms indicates the maximum number of atoms. We use 32 of batch size in EGNN-based PLB and ImagePLB (EGNN) and 8 of batch size on SE3T-based PLB and ImagePLB (SE3T). ImagePLB (EGNN) and ImagePLB (SE3T) indicate that EGNN and SE3T are selected as the encoders of protein pockets, respectively. OOM means out-of-memory on 4090 GPU.

Model	Maximum atoms	GPU Memory
EGNN-based PLB	50	6,190MiB
	100	7,662MiB
	110	8,126MiB
	120	8,472MiB
	130	8,962MiB
	140	9,300MiB
	150	9,644MiB
	200	11,398MiB
ImagePLB (EGNN)	-	8,244MiB
SE3T-based PLB	30	14,968MiB
	40	15,458MiB
	50	16,910MiB
	60	17,984MiB
	70	19,256MiB
	80	21,122MiB
	90	23,322MiB
	100	OOM
ImagePLB (SE3T)	-	14,746MiB

L Time Complexity Analysis

In the LRL module, ResNet18 is used to extract ligand features from multi-view 3D molecular images, where the time complexity of a single convolutional layer is $O(H*W*K^2*C_{in}*C_{out})$. H and W represent height and width of feature maps, respectively. K represents the size of the convolution kernel. C_{in} and C_{out} represent the number of input and output channels. In PRL, EGNN is used to extract protein features from geometry of pocket, where the time complexity of a single EGNN layer is $O(\left|V^P\right|*d*d_{in}^P*d_{out}^P)$. $\left|V^P\right|$ and d represent the number of atoms and degree of the protein pocket, respectively. d_{in}^P and d_{out}^P represent the input and output dimensions of the atom in pocket, respectively. In BRL, the Protein-to-Ligand Fusion Enhancement and Ligand-to-Protein Fusion Enhancement are used to fuse interaction information between ligand and protein, where the time complexity is $O(d_{out}^L*d_{out}^P*\left|V^P\right|)$ and d_{out}^L is the output dimension of ligand. Therefore, the total time complexity of ImagePLB is $O(H*W*K^2*C_{in}*C_{out})+O(\left|V^P\right|*d*d_{in}^P*d_{out}^P)+O(d_{out}^L*d_{out}^P*\left|V^P\right|)$. It is worth noting that we reduce the time complexity of ligand-protein interaction from $O(\left|V^L\right|*\left|V^P\right|)$ to $O(\left|V^P\right|)$, where $\left|V^L\right|$ represents the atom number of ligand, which improves the efficiency of the PLB method when the number of atoms in the ligand is too large. In summary, it can be seen that ImagePLB is independent of the number of atoms and edges of the ligand, which is efficient in predicting the binding of proteins and ligands with a large number of atoms and edges.

References

[1] Hakime Öztürk, Arzucan Özgür, and Elif Ozkirimli. Deepdta: deep drug–target binding affinity prediction. *Bioinformatics*, 34(17):i821–i829, 2018.

[2] Mostafa Karimi, Di Wu, Zhangyang Wang, and Yang Shen. Deepaffinity: interpretable deep learning of compound–protein affinity through unified recurrent and convolutional neural networks. *Bioinformatics*, 35(18):3329–3338, 2019.

[3] Zhi Jin, Tingfang Wu, Taoning Chen, Deng Pan, Xuejiao Wang, Jingxin Xie, Lijun Quan, and Qiang Lyu. Capla: improved prediction of protein–ligand binding affinity by a deep learning approach based on a cross-attention mechanism. *Bioinformatics*, 39(2):btad049, 2023.

[4] Qizhi Pei, Lijun Wu, Jinhua Zhu, Yingce Xia, Shufang Xie, Tao Qin, Haiguang Liu, Tie-Yan Liu, and Rui Yan. Breaking the barriers of data scarcity in drug–target affinity prediction. *Briefings in Bioinformatics*, 24(6):bbad386, 2023.

[5] Jianpeng Cheng, Li Dong, and Mirella Lapata. Long short-term memory-networks for machine reading. In *Proceedings of the 2016 Conference on Empirical Methods in Natural Language Processing*, pages 551–561, 2016.

[6] Ashish Vaswani, Noam Shazeer, Niki Parmar, Jakob Uszkoreit, Llion Jones, Aidan N Gomez, Łukasz Kaiser, and Illia Polosukhin. Attention is all you need. *Advances in neural information processing systems*, 30, 2017.

[7] Shuangye Yin, Elizabeth A Proctor, Alexey A Lugovskoy, and Nikolay V Dokholyan. Fast screening of protein surfaces using geometric invariant fingerprints. *Proceedings of the National Academy of Sciences*, 106(39):16622–16626, 2009.

[8] Xiaolei Zhu, Yi Xiong, and Daisuke Kihara. Large-scale binding ligand prediction by improved patch-based method patch-surfer2. 0. *Bioinformatics*, 31(5):707–713, 2015.

[9] Pablo Gainza, Freyr Sverrisson, Frederico Monti, Emanuele Rodola, D Boscaini, Michael M Bronstein, and Bruno E Correia. Deciphering interaction fingerprints from protein molecular surfaces using geometric deep learning. *Nature Methods*, 17(2):184–192, 2020.

[10] Shiyu Xu, Lian Shen, Menglong Zhang, Changzhi Jiang, Xinyi Zhang, Yanni Xu, Juan Liu, and Xiangrong Liu. Surface-based multimodal protein-ligand binding affinity prediction. *Bioinformatics*, page btae413, 2024.

[11] Vignesh Ram Somnath, Charlotte Bunne, and Andreas Krause. Multi-scale representation learning on proteins. *Advances in Neural Information Processing Systems*, 34:25244–25255, 2021.

[12] Oleg V Tsodikov, M Thomas Record Jr, and Yuri V Sergeev. Novel computer program for fast exact calculation of accessible and molecular surface areas and average surface curvature. *Journal of computational chemistry*, 23(6):600–609, 2002.

[13] Shuxin Wang, Shilei Cao, Dong Wei, Renzhen Wang, Kai Ma, Liansheng Wang, Deyu Meng, and Yefeng Zheng. Lt-net: label transfer by learning reversible voxel-wise correspondence for one-shot medical image segmentation. In *Proceedings of the IEEE/CVF Conference on Computer Vision and Pattern Recognition*, pages 9162–9171, 2020.

[14] Chenyu You, Yuan Zhou, Ruihan Zhao, Lawrence Staib, and James S Duncan. Simcvd: Simple contrastive voxel-wise representation distillation for semi-supervised medical image segmentation. *IEEE Transactions on Medical Imaging*, 41(9):2228–2237, 2022.

[15] Jiageng Mao, Yujing Xue, Minzhe Niu, Haoyue Bai, Jiashi Feng, Xiaodan Liang, Hang Xu, and Chunjing Xu. Voxel transformer for 3d object detection. In *Proceedings of the IEEE/CVF international conference on computer vision*, pages 3164–3173, 2021.

[16] Jinbao Zhang, Jun Liu, Yu Pei, Jingwei Zhang, and Xian Zhao. Learn from voxels: Knowledge distillation for pillar-based 3d object detection with lidar point clouds in autonomous driving. *IEEE Transactions on Intelligent Vehicles*, 2024.

[17] Raphael John Lamarre Townshend, Martin Vögele, Patricia Adriana Suriana, Alexander Derry, Alexander Powers, Yianni Laloudakis, Sidhika Balachandar, Bowen Jing, Brandon M Anderson, Stephan Eismann, et al. Atom3d: Tasks on molecules in three dimensions. In *Thirty-fifth Conference on Neural Information Processing Systems Datasets and Benchmarks Track (Round 1)*, 2021.

[18] Thin Nguyen, Hang Le, Thomas P Quinn, Tri Nguyen, Thuc Duy Le, and Svetha Venkatesh. Graphdta: predicting drug–target binding affinity with graph neural networks. *Bioinformatics*, 37(8):1140–1147, 2021.

[19] Shengchao Liu, Hongyu Guo, and Jian Tang. Molecular geometry pretraining with se (3)-invariant denoising distance matching. In *The Eleventh International Conference on Learning Representations*, 2023.

[20] Fang Wu, Shuting Jin, Yinghui Jiang, Xurui Jin, Bowen Tang, Zhangming Niu, Xiangrong Liu, Qiang Zhang, Xiangxiang Zeng, and Stan Z Li. Pre-training of equivariant graph matching networks with conformation flexibility for drug binding. *Advanced Science*, 9(33):2203796, 2022.

[21] Yuanqi Du, Limei Wang, Dieqiao Feng, Guifeng Wang, Shuiwang Ji, Carla P Gomes, Zhi-Ming Ma, et al. A new perspective on building efficient and expressive 3d equivariant graph neural networks. *Advances in Neural Information Processing Systems*, 36, 2024.

[22] Bowen Gao, Bo Qiang, Haichuan Tan, Yinjun Jia, Minsi Ren, Minsi Lu, Jingjing Liu, Wei-Ying Ma, and Yanyan Lan. Drugclip: Contrasive protein-molecule representation learning for virtual screening. *Advances in Neural Information Processing Systems*, 36, 2024.

[23] Gengmo Zhou, Zhifeng Gao, Qiankun Ding, Hang Zheng, Hongteng Xu, Zhewei Wei, Linfeng Zhang, and Guolin Ke. Uni-mol: A universal 3d molecular representation learning framework. In *The Eleventh International Conference on Learning Representations*, 2023.

[24] Yinhan Liu, Myle Ott, Naman Goyal, Jingfei Du, Mandar Joshi, Danqi Chen, Omer Levy, Mike Lewis, Luke Zettlemoyer, and Veselin Stoyanov. Roberta: A robustly optimized bert pretraining approach. *arXiv preprint arXiv:1907.11692*, 2019.

[25] Alec Radford, Jong Wook Kim, Chris Hallacy, Aditya Ramesh, Gabriel Goh, Sandhini Agarwal, Girish Sastry, Amanda Askell, Pamela Mishkin, Jack Clark, et al. Learning transferable visual models from natural language supervision. In *International conference on machine learning*, pages 8748–8763. PMLR, 2021.

[26] Renxiao Wang, Xueliang Fang, Yipin Lu, Chao-Yie Yang, and Shaomeng Wang. The pdbbind database: methodologies and updates. *Journal of medicinal chemistry*, 48(12):4111–4119, 2005.

[27] Jianyi Yang, Ambrish Roy, and Yang Zhang. Biolip: a semi-manually curated database for biologically relevant ligand–protein interactions. *Nucleic acids research*, 41(D1):D1096–D1103, 2012.

[28] Anna Gaulton, Louisa J Bellis, A Patricia Bento, Jon Chambers, Mark Davies, Anne Hersey, Yvonne Light, Shaun McGlinchey, David Michalovich, Bissan Al-Lazikani, et al. Chembl: a large-scale bioactivity database for drug discovery. *Nucleic acids research*, 40(D1):D1100–D1107, 2012.

[29] Zhihai Liu, Minyi Su, Li Han, Jie Liu, Qifan Yang, Yan Li, and Renxiao Wang. Forging the basis for developing protein–ligand interaction scoring functions. *Accounts of chemical research*, 50(2):302–309, 2017.

[30] Till Siebenmorgen, Filipe Menezes, Sabrina Benassou, Erinc Merdivan, Kieran Didi, André Santos Dias Mourão, Radosław Kitel, Pietro Liò, Stefan Kesselheim, Marie Piraud, et al. Misato: machine learning dataset of protein–ligand complexes for structure-based drug discovery. *Nature Computational Science*, pages 1–12, 2024.

[31] Noel M O'Boyle, Michael Banck, Craig A James, Chris Morley, Tim Vandermeersch, and Geoffrey R Hutchison. Open babel: An open chemical toolbox. *Journal of cheminformatics*, 3:1–14, 2011.

[32] Warren L DeLano et al. Pymol: An open-source molecular graphics tool. *CCP4 Newsl. Protein Crystallogr*, 40(1):82–92, 2002.

[33] Gregory W Kyro, Rafael I Brent, and Victor S Batista. Hac-net: A hybrid attention-based convolutional neural network for highly accurate protein–ligand binding affinity prediction. *Journal of Chemical Information and Modeling*, 63(7):1947–1960, 2023.

[34] Gregory W Kyro, Anthony M Smaldone, Yu Shee, Chuzhi Xu, and Victor S Batista. T-alpha: A hierarchical transformer-based deep neural network for protein–ligand binding affinity prediction with uncertainty-aware self-learning for protein-specific alignment. *Journal of Chemical Information and Modeling*, 65(5):2395–2415, 2025.

[35] Xiangxiang Zeng, Hongxin Xiang, Linhui Yu, Jianmin Wang, Kenli Li, Ruth Nussinov, and Feixiong Cheng. Accurate prediction of molecular properties and drug targets using a self-supervised image representation learning framework. *Nature Machine Intelligence*, 4(11):1004–1016, 2022.

[36] Hongxin Xiang, Shuting Jin, Xiangrong Liu, Xiangxiang Zeng, and Li Zeng. Chemical structure-aware molecular image representation learning. *Briefings in Bioinformatics*, 24(6):bbad404, 2023.

[37] Hongxin Xiang, Shuting Jin, Jun Xia, Man Zhou, Jianmin Wang, Li Zeng, and Xiangxiang Zeng. An image-enhanced molecular graph representation learning framework. In *Proceedings of the Thirty-Third International Joint Conference on Artificial Intelligence*, pages 6107–6115, 2024.

[38] Kai-Yang Zhong, Meng-Liang Wen, Fan-Fang Meng, Xin Li, Bei Jiang, Xin Zeng, and Yi Li. Mmdta: a multimodal deep model for drug-target affinity with a hybrid fusion strategy. *Journal of Chemical Information and Modeling*, 64(7):2878–2888, 2023.

[39] Ding Luo, Dandan Liu, Xiaoyang Qu, Lina Dong, and Binju Wang. Enhancing generalizability in protein–ligand binding affinity prediction with multimodal contrastive learning. *Journal of chemical information and modeling*, 64(6):1892–1906, 2024.

[40] Linlin Zhang, Chunping Ouyang, Yongbin Liu, Yiming Liao, and Zheng Gao. Multimodal contrastive representation learning for drug-target binding affinity prediction. *Methods*, 220:126–133, 2023.

[41] Álvaro González. Measurement of areas on a sphere using fibonacci and latitude–longitude lattices. *Mathematical geosciences*, 42:49–64, 2010.

[42] Travis E Oliphant et al. *Guide to numpy*, volume 1. Trelgol Publishing USA, 2006.
